# AHL quorum sensing regulates T6SS and volatiles production in rice root-colonizing *Enterobacter asburiae* AG129

**DOI:** 10.1093/femsec/fiaf120

**Published:** 2025-12-08

**Authors:** Chandan Kumar, Iris Bertani, Manel Chaouachi, Michael P Myers, Paolina Garbeva, Cristina Bez, Vittorio Venturi

**Affiliations:** International Center for Genetic Engineering and Biotechnology, 34149 Trieste, Italy; International Center for Genetic Engineering and Biotechnology, 34149 Trieste, Italy; International Center for Genetic Engineering and Biotechnology, 34149 Trieste, Italy; International Center for Genetic Engineering and Biotechnology, 34149 Trieste, Italy; Department of Microbial Ecology, The Netherlands Institute of Ecology (NIOO-KNAW), Droevendaalsesteeg 10, 6708 PB Wageningen, The Netherlands; International Center for Genetic Engineering and Biotechnology, 34149 Trieste, Italy; International Center for Genetic Engineering and Biotechnology, 34149 Trieste, Italy; African Genome Center, University Mohammed VI Polytechnic (UM6P), 43150 Ben Guerir, Morocco

**Keywords:** endosphere/rhizosphere, *Enterobacter asburiae* AG129, *N*-butanoyl homoserine lactone (C4-AHL), quorum sensing, Type VI secretion system (T6SS), volatile organic compounds (VOCs)

## Abstract

Pseudomonadota (formerly Proteobacteria) commonly use a contact independent cell–cell communication system known as quorum sensing (QS) mediated by *N*-acyl-homoserine lactone (AHL) signal molecules. The canonical AHL QS system involves a *luxI*-family gene, which encodes an AHL synthase, and a *luxR*-family gene, which encodes a transcriptional regulator responsive to the cognate AHL(s). This study involves the AHL QS system of *Enterobacter asburiae* AG129, a root associated strain isolated from rice (*Oryza sativa*). *Enterobacter asburiae* AG129 produces the *N*-butanoyl homoserine lactone (C4-AHL) signal molecule. Genome sequencing of strain AG129 revealed the presence of a canonical AHL QS system, comprising genetically adjacent *easI*-like and *easR*-like genes. A genomic *easI* knockout mutant was no longer able to produce AHLs, but the in-trans complementation with a plasmid carrying the *easI* gene restored the AHL production. QS mediated by AHLs in AG129 was found to influence rice root colonization, and secretome analysis highlighted a significant regulatory role in the expression of Type VI secretion system (T6SS) proteins. Gas chromatography–mass spectrometry analysis identified 16 volatile organic compounds (VOCs) that were more abundantly emitted by the wild-type strain compared to the *easI* mutant. Overall, our findings suggest that AHL-based QS in *E. asburiae* AG129 positively regulates T6SS expression and VOC production, while negatively affecting root colonization and motility. This study is among the first to explore the role of QS signaling in a bacterial root-endophyte, providing evidence of a connection between QS activity and the ability of the bacterium to inhabit, compete and colonize the plant root endosphere.

## Teaser

This study reveals that AHL-mediated QS in *E. asburiae* AG129 regulates T6SS and VOCs production, while impairing root colonization in rice, highlighting its role in plant–endophyte interaction. Figure created with BioRender.

## Introduction

One of the most studied contact independent cell–cell communication systems in bacteria, known as quorum sensing (QS), was first reported in the regulation of bioluminescence in response to cell density in the marine bacterium in *Aliivibrio* (formally *Vibrio*) *fischeri* (Fuqua et al. 1994: 269–75, Nealson et al. 1970: 313–22). QS in bacteria involves the production and detection of signal molecules to regulate the expression of target genes associated with community behaviors, including symbiosis, root colonization, exopolysaccharide production, virulence, motility, biofilm formation, nodulation, and the secretion of various compounds such as antibiotics, toxins, surfactants, extracellular enzymes, and secondary metabolites (Atkinson and Williams 2009: 959–78, Barnard et al. 2007: 1165–83, Rosemeyer et al. 1998: 815–21, Schuster et al. 2017: 885). QS is used by both beneficial plant-associated and plant pathogenic bacteria. In beneficial microbes, QS promotes plant health, by coordinating symbiotic relationships, and the production of compounds that protect the plant, whereas in phytopathogenic bacteria it facilitates disease progression by regulating the production of toxins, cell wall-degrading enzymes, and other factors that enable breakdown of plant tissues and evasion of plant immune responses (Venturi and Keel 2016: 187–98).

In Pseudomonadota, the most common QS system is mediated by *N*-acyl-homoserine lactones (AHLs) signal molecules (Wellington and Greenberg 2019: 10.1128/mbio. 00146–19). AHL signals share a conserved lactone core, but vary in the acyl moiety, which can be a fatty acid ranging from 4 to 20 carbons long, with potential oxidation on the C3 carbon and varying degrees of unsaturation, or can have an aromatic or branched structure. These structural variations confer specificity and selectivity to the system in different bacterial species (Ampomah-Wireko et al. 2021: 113 864, McInnis and Blackwell 2011: 4820–8, Pearson et al. 1999: 1203–10). The canonical AHL QS system involves two key components: a *luxI*-family gene, which encodes the enzyme responsible for synthesizing AHLs (AHL synthase), and a *luxR*-family gene, which encodes a transcriptional regulator that detects and binds the cognate AHLs. Commonly, when AHLs reach a threshold concentration, they bind to the LuxR regulator, which then regulates the transcription of target genes (Fuqua and Greenberg 2002: 685–95, Rutherford and Bassler 2012: a012427).

Endophytic bacteria inhabit the internal tissues of plants, such as roots, stems, and leaves, without harming the host (Compant et al. 2010: 669–78, Reinhold-Hurek and Hurek 2011: 435–43). Endophytes enter the inner tissues of plants in several ways, including via natural openings like stomata and lenticels, root hairs, wounds, direct penetration, emerging roots, and seed transmission (Mengistu 2020: 6 927 219, Rajesh and Rai 2014: 290–5). Once established inside the plant, many endophytes form mutualistic relationships that enhance plant health by promoting stress tolerance, nutrient acquisition, immune modulation, rhizosphere colonization, and overall plant fitness (Berg et al. 2014: 491, Bulgarelli et al. 2013: 807–38, Hardoim et al. 2015: 293–320, Mengistu 2020: 6 927 219, Schuster et al. 2017: 885). Some endophytic bacteria have been reported to produce QS signaling molecules, volatile organic compounds (VOCs) and phytohormones which could play a role in plant–bacteria interaction (Hardoim et al. 2015: 293–320, Santoyo et al. 2016, Hartmann et al. 2024: 92–9). Despite its potential significance, our understanding of the presence, distribution, and functional role of AHL-mediated QS systems in endophytic bacteria remains very limited. Investigating QS in endophytes could uncover key regulatory mechanisms that underlie beneficial traits such as enhanced plant growth, stress resistance, and disease suppression. Moreover, understanding how QS governs the behavior of endophytes within plant tissues could offer new strategies for sustainable agriculture, such as the development of bioinoculants that improve crop resilience and productivity.

*Enterobacter asburiae* is a beneficial endophytic bacterium known to colonize a wide range of plant species, including maize, cotton, sugarcane, rice, and legumes (Ogbo and Okonkwo 2012: 368–74, Singh et al. 2020: 600 417). As a plant growth-promoting bacterium, it enhances plant development through multiple mechanisms such as indole-3-acetic acid (IAA) production, phosphate solubilization, nitrogen fixation via nitrogenase activity, siderophore production, and ACC deaminase activity that mitigates ethylene-related stress (Dimaria et al. 2024: 127 833, Ning et al. 2024: 333–49, Zhang et al. 2025). Several strains have shown effects in different crops, for example strain BY4 contributed up to 35% of nitrogen to sugarcane through biological nitrogen fixation (Singh et al. 2020: 600 417); strain D2 improved rice growth and salt tolerance by modulating ion homeostasis and synthesizing growth-promoting metabolites (Ning et al. 2024: 333–49); and strain HK169 exhibited biocontrol activity against root-knot nematodes, reducing gall formation by 66% and increasing plant biomass (Saikia et al. 2023).

Despite its beneficial plant-associated roles, *E. asburiae* has also been described as an opportunistic human pathogen. It has been isolated from clinical settings, including cases of pneumonia, wound infections, urinary tract infections, and bacteremia, particularly in immunocompromised patients (Mattioni Marchetti et al. 2024: 6220). These dual lifestyles suggest that *E. asburiae* harbors versatile genetic and regulatory systems enabling adaptation to both plant and human hosts. Understanding the QS as possible regulatory switch, balancing colonization, biofilm formation and secondary metabolisms secretion across different niches, may help elucidate the molecular mechanisms underlying the ecological versatility and host adaptability of *E. asburiae*.

The aim of this study was to characterize the AHL QS system present in the rice endophyte *Enterobacter asburiae* AG129 (Bertani et al. 2016: 388–98). The research included proteomic analyses, *in planta* experiments, and profiling of secreted volatile metabolites. Our results showed that AHL-mediated QS in endophytic *E. asburiae* plays a crucial role in plant colonization, bacterial competition through Type VI secretion (T6SS), and the regulation of VOC production. This study offers new insight into the role of QS in shaping the lifestyle of endophytic bacteria, highlighting its involvement in the ecological interactions between members of the endophytic community and between plants and their associated endophytes.

## Materials and methods

### Bacterial strain and growth conditions

The bacterial strain and plasmids used in this study are listed in Table S1. Bacterial strain *E. asburiae* strain AG129 was isolated from a rice cultivar grown in Italy (Bertani et al. 2016: 388–98). Bacterial strains were grown in either Luria–Bertani (LB) broth/agar, tryptic soy (TS) or artificial guttation fluid media (AGF- 10.5 g/l K_2_HPO_4_, 4.5 g/l KH_2_PO_4_, 1 g/l (NH_4_)_2_SO_4_, 1 mM MgSO_4_, 10 mM L-glutamine, 6 mM L-histidine, and 4 mM L-aspartic acid, adapted from previously protocols at 30°C or 37°C (Ryan et al. 2007: 429–42). In order to obtain rifampicin (Rif) resistant *E. asburiae* AG129, cells were grown in LB agar plates supplemented with Rif 100 µg/ml^−1^. When required, antibiotics for the growth of tested strains were added at the following concentrations: nitrofurantoin (Nf) 100 µg ml ^−1^, rifampicin (Rif) 100 µg ml^−1^. The mutants of each strain (carrying a knock-out mutation) have been grown using 100 µg ml^−1^ kanamycin (Km) as an antibiotic for the selection. The complemented strains have been grown using 100 µg ml^−1^ kanamycin (Km) and tetracycline (Tc) 40 µg ml^−1^. *Escherichia coli* DH5α was routinely grown at 37°C in LB broth and antibiotics were added when required at the following concentrations: gentamycin (Gm) 10 µg ml^−1^, tetracycline (Tc) 10 µg ml^−1^.

### Genome sequencing of *Enterobacter asburiae* AG129

*Enterobacter asburiae* AG129 was grown overnight in TS broth at 30°C; the culture was then centrifuged at 6000 RPM for 5 min; the cells were collected, and genomic DNA was subsequently purified using the Norgen Biotek Corp bacterial Genomic DNA Isolation Kit (Schmon Parkway, Thorold, ON, L2V 4Y6, Canada). The genome was sequenced with the Illumina NovaSeq 6000 platform using 150 bp paired-end and following the tagmentation Illumina Nextera XT protocol (Illumina Inc., San Diego, CA, USA). The assembly was performed with Unicycler v. 0.5.0 and the assembly statistics were recorded with QUAST v. 5.2.0 (Gurevich et al. 2013: 1072–5). The assembled genome was then loaded in the Integrated Microbial Genomes and Metagenomes (IMG/M) database under analysis project ID Ga0485092 and automatically annotated, using annotation pipeline IMG Annotation Pipeline v.4.16.6 (Markowitz et al. 2009: 2271–8). The genome was also submitted and annotated using RAST (Rapid Annotation using Subsystem Technology) Server (Aziz et al. 2008: 1–15). The functional annotation as well as the phylogenetic characterization were determined by DFAST (Tanizawa et al. 2018: 1037–9) ran from https://dfast.ddbj.nig.ac.jp/.

### Construction of *E. asburiae* AG129 *easI* deletion mutant and its genetic complementation

For the deletion of *easI* gene, the upstream region of the gene was amplified using the pair of primers luxIBfw and luxIpRw2, while the downstream gene region was amplified using the primer luxIPfw3 and luxIBRw. These two fragments were then combined by fusion PCR using primers luxIBfw and luxIBRw, resulting in the deletion of 301 nucleotides of the *easI* gene and the replacement with a *Pst*I restriction site. The fragment was then ligated into pGEM-T easy (Promega) per manufacturer’s instructions and the kanamycin (Km^R^) gene cassette originally derived from pUCK4 (Pharmacia Biotech) Table S1 was cloned in the *Pst*I restriction site. The final construct was excised and cloned in the *Bam*HI site of the pLAFR3-Tc^R^ plasmid. The pLAFR3*easI*:: kan was then introduced into strain AG129Rif^R^ via triparental conjugation. Plasmid pPH1JI was conjugated, which belongs to the same compatibility group as pLAFR3-Tc^R^ and transconjugants were selected for Rif^R^, Km^R^, Gm^R^, and checked for Tc^S^. This process allowed the selection of an *easI* knock-out mutant hereafter named as AG129*easI* (verified via PCR).

In order to construct the *easI* complementation plasmid, the *easI* gene was amplified together with 5′ and 3′ flanking region (200 nucleotides each part) from genomic DNA as template with the forward primers *LuxI*comFK and reverse primer *LuxI*compRX. The sequence was verified by DNA sequencing and the fragment was cloned into plasmid pBBR1MCS-3 under the transcription of the pLac promoter of pBBR1MCS-3 yielding pBBR1MCS-3*easI*. This plasmid was conjugated in the mutant strain AG129*easI* and selected for Rif^R^, Km^R^, and Tc^R^ yielding AG129*easI*(pBBR1MCS-3*easI*) which is the *easI* mutant strain complemented.

### AHLs detection

The AG129 strain was grown in liquid medium for 18 hr and the supernatant was recovered by centrifugation (6000 RPM for 5 min); the spent media was filtered (0.45 µm pore diameter). Six hundred microliters of media were acidified with 13 µl of 1 M formic acid, mixed 1:1 with water-saturated ethyl acetate, shaken for 30 min, and the organic phase was collected.

For tandem mass spectrometry (MS–MS), extracts were reconstituted in 100 µl of 0.1% formic acid. Ten microliters were injected onto a column and developed with a 5%–80% acetonitrile gradient. Eluted compounds were analyzed using a 6550 QTOF, with quantitation based on precursor to product ion conversion. AHLs were quantified via peak area comparison to standards using MSHunter software (Špacapan et al. 2024: e04179-23).

### *In vitro* phenotype assays

Several *in vitro* phenotype assays were performed in the following media/conditions: swarming (tryptic soy with 0.5% agar) and swimming (tryptic soy with 0.3% agar), proteolytic activity [3 g TSB + 8 g of Agar in 500 ml mix + 5 g (1%) Skim milk], lipolytic activity [3 g TSB + 8 g of Agar in 500 ml mix and autoclave 5 ml (1%) tributyrin mixed with 10 ml of warm TSA], phosphate solubilization (NBRIP medium), EPS (exopolysaccharides) production (YEM medium), IAA production (salkowski reagent), and QS-AHLs synthesis [plate T-Streak next to the QS sensor *Chromobacterium violaceum* CV026 (Fig. S2) (McClean et al. 1997: 3703–11)].

### Rice root rhizosphere and endosphere colonization experiments

Rice seeds were surface sterilized using 10% bleach for 10 min, followed by 70% ethanol for 1 min, and then rinsed five times with sterile water. Sterilized seeds were then pre-germinated for 7 days before seedling inoculation. The colonization experiment was carried out with AG129(pBBR1MCS-3) WT, AG129*easI*(pBBR1MCS-3) and with AG129*easI*(pBBR1MCS-3*easI*). Before the colonization experiments the pBBR1MCS-3 was introduced by triparental conjugation in the AG129 WT and the AG129*easI* strains to uniform the detection and the counting of the respective inoculated strains. WT, *easI*-mutant, and *easI*-complemented bacterial strains were grown overnight, the optical density at 600 nm (OD_600_) was measured and adjusted to OD_600_ 1.0 for all strains. Seven-day-old pre-germinated seedlings were then submerged in bacterial cultures for 1 hr. Inoculated seedlings were placed in 50-ml Falcon tubes containing semi-solid Hoagland solution (Steindler et al. 2009: 5131–40) with 0.4% agar and grown for 7 days under controlled conditions (30°C, 16-hr photoperiod). Seven days post-inoculation, plants were harvested and roots were rinsed with sterile water to remove the Hoagland solution agar attached.

Clean roots were then weighed and transferred to 8 ml of 1X PBS (Phosphate Buffered Saline) and vortexed for 10 min to recover rhizoplane-adhered bacterial cells. After the recovering of the rhizosphere, the roots were surface sterilized following the method described previously (Bertani et al. 2016: 388–98)and then macerated in 2 ml of PBS in order to recover the root endosphere. For both rhizosphere and endosphere colonization counting, serial dilutions of the recovered bacterial suspension were plated on LB medium containing the respective antibiotics: AG129(pBBR1MCS-3) WT, was plated on Rif 100 µg ml^−1^ and Tc 40 µg ml^−1^, the AG129*easI*(pBBR1MCS-3) mutant plated on Rif 100 µg ml^−1^, Km 100 µg ml^−1^, and Tc 40 µg ml^−1^, while the complemented strain AG129*easI*(pBBBR1MCS-3*easI*) was plated on Rif 100 µg ml^−1^, Km 100 µg ml^−1^, and Tc 40 µg ml^−1^. Plates were then incubated overnight, and colony-forming units (CFUs) were counted to determine bacterial colonization expressed as CFU per gram of root.

### Secretome profiling

To determine the extracellular proteome secreted by the AG129 WT and the AG129*easI* mutant, both strains were grown in plant mimic AGF liquid media following a modified methodology as previously described protocols (Mosquito et al. 2020: 349–63). Briefly, 50 ml cultures were grown in AGF media at 30°C for 16 hr until they reached a pre-stationary phase with an OD_600_ of 1.2. The cultures were then centrifuged at 2000 RPM for 10 min at 4°C to collect the supernatant. This centrifugation step was further repeated, to ensure thorough removal of bacterial cells, and followed by filtration of the supernatant through a 0.45 µm membrane to eliminate any remaining bacterial residues. The proteins in the supernatant were precipitated by adding trichloroacetic acid to a final concentration of 10% (wt/vol), and the samples were centrifuged to recover the protein pellets. These pellets were reconstituted with trimethyl ammonium bicarbonate buffer followed by trypsin digestion and subsequently used for liquid chromatography mass spectrometry (LC/MS) acquisition. Protein pellets were resuspended in 100 mM Triethyl Ammonium Bicarbonate (Sigma) supplemented with 0.2% Protease Max (Promega) by vortexing. The samples were then reduced by the addition of tris(2-carboxyethyl)phosphine (Sigma) to a final concentration of 10 mM and heated to 65°C for 5 min. After cooling to r.t., the samples were alkylated by the addition of acrylamide (Biorad) to 20 mM and a further incubation for 1 hr at r.t. The samples were digested by the addition of 0.5 ug of trypsin (Promega) at 37°C for 16 h. The digestion was stopped by the addition of formic acid (Sigma) to 0.1%. The digestions were cleaned up using STAGE tips (Rappsilber et al. 2007: 1896–906). The digests were resupended in 10 μl of 0.1% formic acid and analyzed by LC–MS–MS using a 0.1 × 20 cm column packed with 2.7 um Bioshell peptide resin (Sigma). The column was developed by a 60 min discontinous gradient from 3% to 80% of Acetonitrile. The effluent was directed to the source of an Agilent 6550 mass spectrometer with 20 MS–MS scans following each survey scan. Raw LC–MS–MS data were first converted to mzML format using ProteoWizard msconvert and processed for peptide-identification. Initial searches were carried out with the X!Tandem search engine (Craig and Beavis 2004: 1466–7) against the Uniprot protein database for *Enterobacter asburiae* (taxon 61 645) and filtered at an FDR of <1%. Searches were performed with trypsin specificity (allowing two missed cleavages), precursor and fragment tolerances of 10 ppm and 0.4 Da, respectively, carbamidomethylation of cysteine as a fixed modification, and oxidation of methionine and N-terminal acetylation as variable modifications. To obtain quantitative information, all raw files were subsequently analysed through the FragPipe pipeline. Prior to statistical analysis, contaminant entries were removed, and proteins not consistently identified or quantified within the same experimental condition were excluded. MaxLFQ intensity values were log_2_-transformed, and samples were grouped according to their biological conditions. Missing values were imputed under a Missing-Not-At-Random (MNAR) assumption using random draws from a left-shifted Gaussian distribution (1.8 standard deviations downshift, width 0.3). Differential protein abundance was assessed using protein-wise linear models combined with empirical Bayes moderation as implemented in the limma package (Bioconductor). Pairwise comparisons, specifically delta versus wild type, were evaluated by applying a Benjamini–Hochberg adjusted p-value cutoff of 0.05 together with an absolute log_2_-fold-change threshold of ≥1 to define significantly regulated proteins. The complete FragPipe Analyst report is available as File S2.

### Gene promoter constructs and β-galactosidase assays

Based on the results from the secretome analysis, and to further validate the role of *easI* in regulating T6SS loci, three gene promoters were selected for detailed study. These promoters were located upstream of genes encoding a hypothetical protein (Hyp1), a T6SS-secreted protein (Hcp), and another hypothetical protein (Hyp6).

The promoter construct for hyp1 was 1101 bp long and included XbaI and KpnI restriction sites at the 5′ and 3′ ends, respectively. The hcp promoter construct was 287 bp, also flanked by XbaI and KpnI sites, while the hyp6 promoter construct was 694 bp, with PstI and KpnI restriction sites at the 5′ and 3′ ends. All three promoter sequences were synthesized by GenScript Biotech (Rijswijk, Netherlands) and initially cloned into the pUC57-Amp^R^ vector (Table S2).These fragments were excised from the pUC57-Amp^R^ vector using the restriction sites mentioned above and cloned into the corresponding sites of the pMP220-Tc^R^ promoterless vector (Spaink et al. 1987: 27–39). Correct insertion was verified by PCR using the primers listed in Table S2. The resulting constructs were conjugated into both the AG129 WT strain and the AG129 *easI* mutant, generating the strains AG129(pMP220*hyp1*), AG129(pMP220*hcp*), AG129(pMP220*hyp6*), AG129*easI*(pMP220*hyp1*), AG129*easI*(pMP220*hcp*), and AG129*easI*(pMP220*hyp6*). The pMP220-Tc^R^ empty vector was also introduced into both AG129 WT and AG129*easI* strains as controls. Promoter activities were then assessed using β-galactosidase Miller assays (Mattiuzzo et al. 2011: 145–62).For chemical complementation, 1 µM of C4-AHL was added to the culture of AG129(pMP220*hyp1*), AG129(pMP220*hcp*), AG129(pMP220*hyp6*), AG129*easI*(pMP220*hyp1*), AG129*easI*(pMP220*hcp*), and AG129*easI*(pMP220*hyp6*).

### Bacterial contact dependent killing assays

The assay was performed with modifications based on a previously described method (Rezzonico et al. 2012: 6645–65). Three biological replicates were conducted for each experimental condition. The CFU/ml value for each biological replicate represents the mean of five technical replicates. Briefly, *E. asburiae* AG129 WT, the easI knockout mutant (AG129ΔeasI), the complemented strain (AG129ΔeasI:: easI), and the target *Escherichia coli* DH5α strain were grown overnight in LB medium. Cultures were adjusted to an optical density of OD600 = 1. Equal volumes (10 μl each) of normalized *E. coli* and either AG129 WT, AG129*easI* and complemented strain were mixed at a 1:1 ratio, and 20 μl of the mixture was spotted onto LB agar plates, followed by incubation at 30°C. Samples were collected from the plates at three time points: 0 hr, 4 hr, and 18 hr. Cells were then resuspended in 1 ml of LB.

In parallel, a liquid co-culture was prepared by inoculating the 1:1 mixed bacterial suspension into 10 ml of LB medium and incubating it with shaking at 30°C. Samples were taken at 0 hr and 18 hr.

For both solid and liquid co-culture conditions, collected samples were serially diluted and plated onto LB agar supplemented with nalidixic acid 20 µg ml^−1^ to selectively count *E. coli* CFUs, since AG129 WT, AG129*easI* and the complemented strain are sensitive to nalidixic acid and plated onto LB agar supplemented with rifampicin 100 µg ml^−1^ to selectively count *Enterobacter* CFUs. As controls, 10 μl of *E. coli* and *Enterobacter* strains grown alone under both solid and liquid conditions were processed similarly.

### Bacterial volatile compounds (VOCs) trapping and VOCs identification

In order to trap bacterial volatiles, a two-compartment setup was prepared using glass Petri dishes. One compartment was filled with LB agar, while the other was left empty. Freshly grown cultures of AG129 WT and AG129*easI* were spread onto the LB agar and incubated at 30°C to allow for bacterial growth. After 16 hr of incubation, the sorbent material HiSorb probe (model H1-AXABC, Markes International Ltd, Llantrisant, UK) was placed in the empty compartment of the Petri dish, which was then sealed with lid and parafilm. The HiSorb probes were preconditioned at 280°C for 1 hr using a U-CTE micro-chamber/thermal extractor (Markes International Ltd., Llantrisant, UK) prior application.

The dishes were incubated for an additional 5 hr at 30°C to trap the volatiles produced by AG129 WT and AG129*easI*. A Petri dish containing only LB agar without bacterial culture served as a control. For each condition (control, AG129 WT and AG129*easI*) 4 plates were used. The HiSorb were recovered and the trapped volatiles were subsequently analyzed using gas chromatography coupled with quadrupole time-of-flight mass spectrometry (GC/Q-TOF). The VOCs were desorbed from from HiSorb probes using an automatic desorption unit (Unity TD-100, Markes International, Llantrisant, UK) with the helium gas at 50 ml min^−1^ at the temperature of 240°C for 8 min. The released volatile compounds were then trapped with a cold trap at −10°C and reheated at 280°C for 5 min. The volatiles were then transferred splitless or with split 1:9 (280°C transfer line) to the GC/Q-TOF (model Agilent 7890B GC and the Agilent 7200A Q-TOF, Santa Clara, CA, USA) with an DB-5 ms ultra-inert column (30 m length, 0.25 mm internal diameter, 0.25 μm film thickness, Agilent Technologies, Inc., Santa Clara, CA, USA) with a run time of 35.6 min and a flow of 1.2 ml/min (constant flow). The temperature program was set to 39°C for 1 min followed by heating up to 315°C with 10°C/min and holding for 7 min. Then, volatile compounds were detected by the GC/Q-TOF system running at 70 eV in electron ionization (EI) mode with a temperature source of 230°C. The mass spectra of the volatile compounds were acquired in full-scan-mode (m/z 30–400, 4 scans/s, 2 GHz Extended Dynamic Range). Volatile analyses and identification was performed as previously described (Lee Díaz et al. 2022: 1612).

### Antifungal and antibacterial activity of *Enterobacter* VOCs

The antifungal activity of *E. asburiae* WT, *easI*, and complemented strain was assessed using double-compartment Petri dishes. In the first compartment, 5-mm mycelial plugs of *Botrytis cinerea* strain BC1 was placed onto PDA medium. *Enterobacter* strains were grown overnight in TSB at 30°C, and cultures were adjusted to an optical density (OD_600_) of 1. A volume of 100 µl of each *Enterobacter* strain was streaked onto the medium in the second compartment. After 7 days of incubation at 25°C, the diameters of fungal strains were measured to evaluate inhibition by bacterial VOCs. To assess VOC-mediated antibacterial activity, *Enterobacter* strains and the pathogenic strain *Dickeya zeae* DZ2Q were grown overnight in TSB at 30°C. Cultures were adjusted to an OD_600_ of 1. In the first compartment of the double-compartment Petri dish, 100 µl of each *Enterobacter* strain was streaked. In the second compartment, 20 µl of *D. zeae* was spotted onto TSA medium. After 48 hr of incubation, the diameter of the *D. zeae* colony was measured, and the spot was subsequently recovered for serial dilution and viable cell counting.

## Results

### *Enterobacter asburiae* strain AG129 displays several plant associated phenotypes *in vitro*

*Enterobacter asburiae* strain AG129, isolated as an endophyte from rice cultivated in Italy (Bertani et al. 2016: 388–98), was evaluated for several *in vitro* traits commonly associated with plant growth promotion (PGP) and plant colonization abilities. These included phosphate solubilization, production of exopolysaccharides (EPS), IAA synthesis and proteolytic and lipolytic activity (Table S3). *Enterobacter asburiae* AG129 exhibited solubilizing phosphate ability, production of EPS, and synthesized IAA, although it showed no detectable proteolytic and lipolytic activity under the tested conditions.

### *Enterobacter asburiae* strain AG129 possesses one AHL QS system

**Figure 1.**
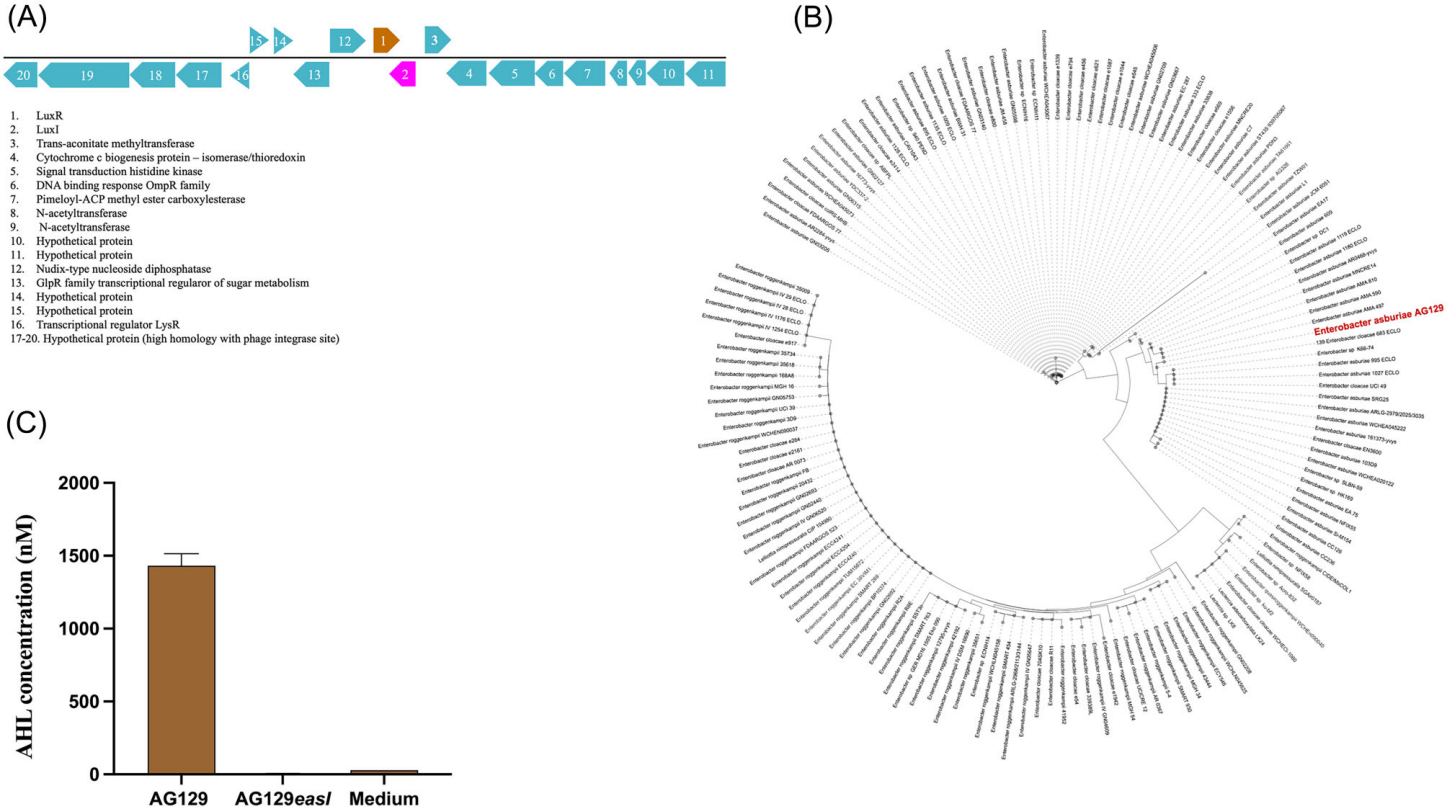
Presence of AHL QS system in *Enterobacter asburiae* AG129; A) Genetic map of the complete *easI/R* AHL QS system present in the *E. asburiae* AG129. Each number corresponds to the gene. B) Phylogenetic tree representing *easI/R* and the neighborhood region conservation among *Enterobacter spp*. Bacterial Genomes from IMG database were searched for presence of *easI/R* genes and conserved genetic context using the “top homolog” search feature in JGI IMG. The tree of the EasI/R representatives was made by aligning their protein sequences using Clustal omega with standard settings (Sievers et al. 2011: 539), and then using FastTree with the alignment as input with standard settings (Price et al. 2009: 1641–50). The tree is visualized and annotated using iTOL (Letunic and Bork 2021: W293-W6). C) Identification of short-chain C4-AHL produced by AG129 after 24 hr of growth via LC-MS. Graph shows averages and standard error of means; and AHL in nM concentration.

*Enterobacter* spp. have been previously reported to possess AHL QS systems (Lau et al. 2013: 14189–99, Rajesh and Rai 2014: 290–5, Rezzonico et al. 2012: 6645–65). In order to determine if *E. asburiae* AG129 contained an AHL QS system, its genome was sequenced and the functional annotation as well as the phylogenetic characterization were determined (Tanizawa et al. 2018: 1037–9) (Table S4). Genome mining revealed the presence of a complete AHL QS system consisting of a *luxI*-family and of *luxR-*family homologs to *easI/R*, which is an AHL QS system previously reported in *E. asburiae* (Lau et al. 2018: e00610, Tanizawa et al. 2018: 1037–9). The *easI* and *easR* genes were located adjacent to each other oriented in opposite directions (Fig. 1A). Comparative genome analysis, performed on JGI/IMG database, revealed that the *easI/R* locus and its surrounding regions are conserved across 154 genomes, including multiple *Enterobacter* species (*asburiae, cloacae, roggenkampii, quasiroggenkampii*) and related genera within the *Enterobacteriaceae* family, such as *Leclercia* and *Lelliottia* (Fig. 1B).

### The *E. asburiae* AG129 EasI/R AHL QS system produces C4-AHL

*Enterobacter asburiae* AG129 was found to produce AHLs, as demonstrated by violacein induction in a plate assay using the AHL biosensor *Chromobacterium violaceum* CV026 (Fig. S2). To investigate the QS system, an *easI* knock-out mutant and the corresponding complemented strain were generated. When T-streaked adjacent to CV026, only the wild-type and complemented strains induced violacein production, confirming that AHL synthesis is dependent on *easI* (Fig. S2). To identify the specific AHL produced by EasI, cultures were grown in LB medium for 24 hr, and AHLs were extracted and analyzed by LC–MS. The wild-type strain was found to produce *N*-butanoyl homoserine lactone (C4-AHL), whereas the mutant strain did not, confirming EasI as the C4-AHL synthase in *E. asburiae* AG129 (Fig. 1C; Table S5).

### Role of the AHL QS system *in planta* colonization and PGP *in vitro* activities

**Figure 2.**
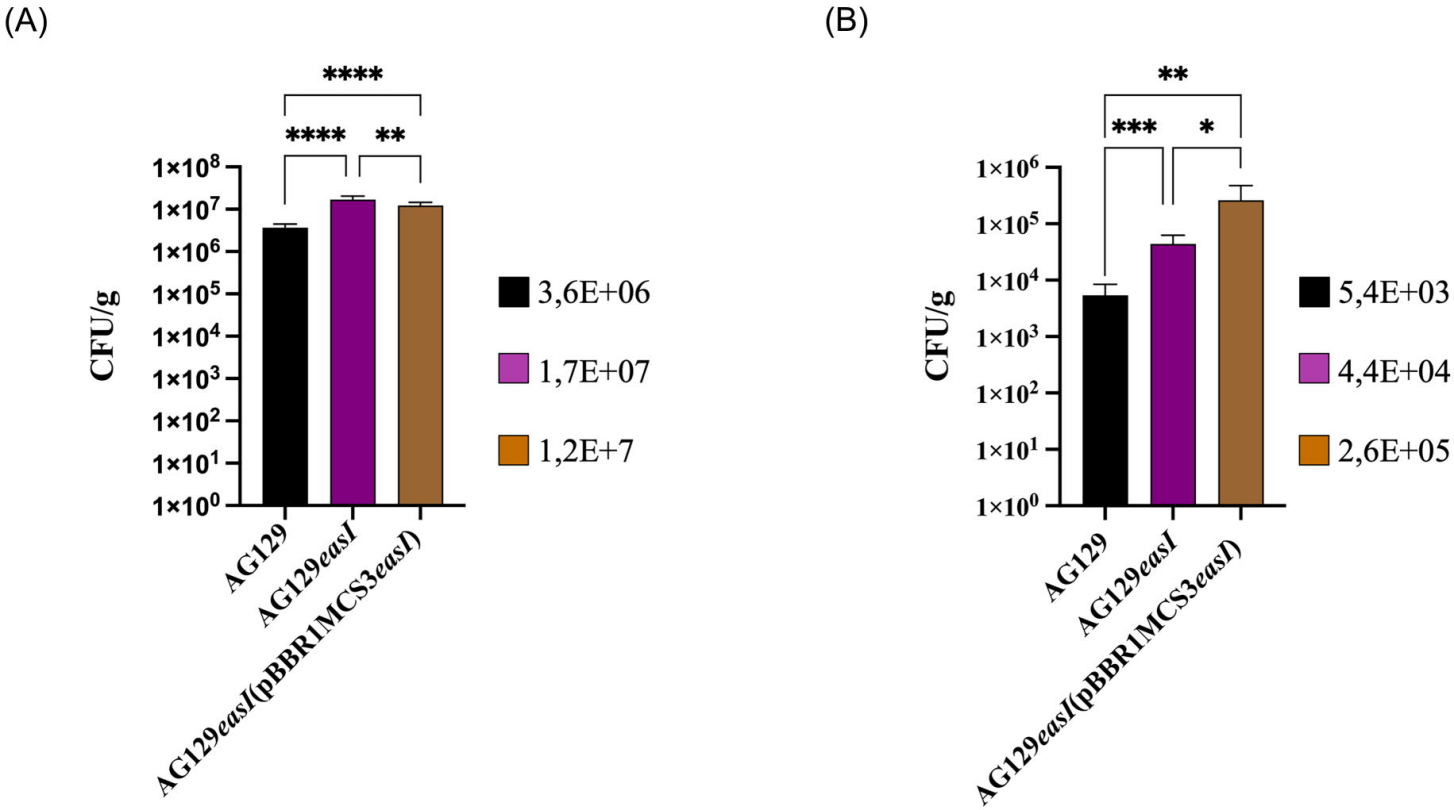
*In planta* colonization experiment testing AG129 WT, AG129*easI* and AG129*easI*(pBBR1MCS3*easI*). Ten plants (*n* = 10) were used in each group of inoculations. A) Quantification of CFUs/g of root in the rhizosphere 7 days post inoculation. B) Quantification of CFUs/g of root in the endosphere 7 days post inoculation. To assess statistical significance, *t*-test was used. All data are presented as means ± standard error (SD). The error bars indicate standard deviations. GraphPad Prism 9.2 (GraphPad Software, Inc.) was used for statistical analysis. *P < 0.05 **P < 0.005, ***P < 0.0005, ****P < 0.00005 as indicated.

It was of interest to determine the role of AHL QS in the colonization of the rice rhizosphere and root endosphere. The results showed that the AG129*easI* mutant exhibited statistically significant higher colonization in both the rhizosphere (1.7E+07 CFU/g) and endosphere (4.4E+04 CFU/g) plant compartment compared to the wild-type AG129 (3.6E+06 CFU/g and 5.4E+03 CFU/g, respectively). The complemented strain partially restored colonization levels to those observed in the wild type (1.2E+07 CFU/g in the rhizosphere; 2.6E+06 CFU/g in the endosphere) (Fig. 2A, 2B). These findings suggest that the AHL QS system negatively regulates bacterial colonization of rice roots. To assess whether AHL QS also influences other *in vitro* growth-promoting traits, the same plant-associated phenotypes described above were determined in the *easI* mutant and complemented strain. Results revealed no statistically significant differences among the tested phenotypes, suggesting that under the conditions used here, the QS system does not play a regulatory role in these plant growth-related traits.

### Comparative secretome analysis of *E. asburiae* AG129 WT and the *easI* mutant

It was of interest to determine the proteins produced and secreted in the extracellular medium under AHL QS regulation in strain AG129 as these could be involved in bacterial behavior and environmental interactions. A secretome study was performed analyzing proteins secreted by AG129 WT and AG129*easI* mutant. Bacterial cultures were grown in plant mimic medium, and the supernatant was used for extracellular secretome analysis by LC-MS as described in the Materials and Methods section.

**Figure 3.**
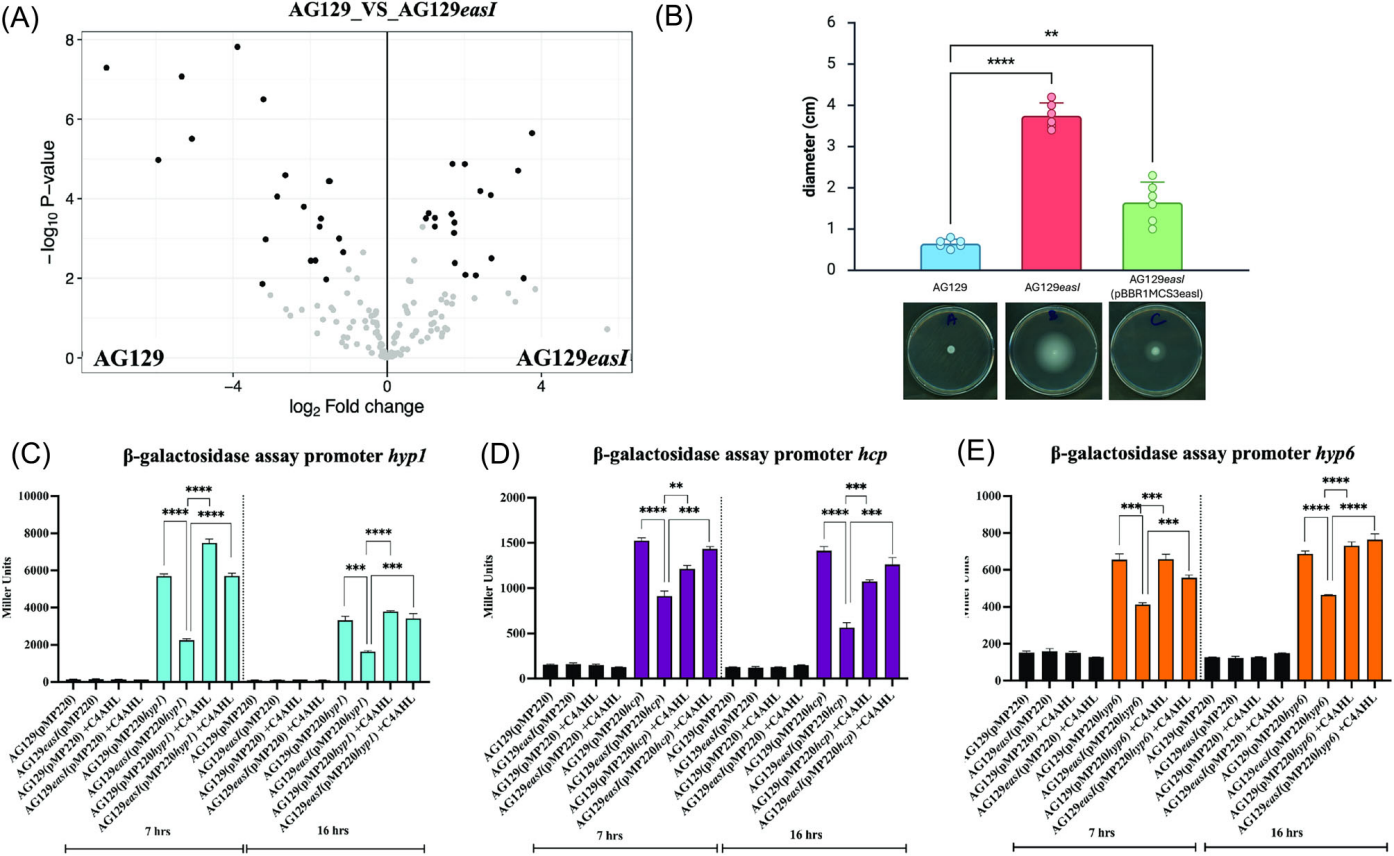
Secretome profiles of *E. asburiae* AG129 wild type and AG129*easI* mutant reveal AHL-QS regulation of T6SS. A) Volcano plot summarizing the differentially abundant secreted proteins between the AG129 wild type and AG129*easI* mutant. The *x*-axis shows the log_2_ fold change, indicating the magnitude of protein regulation (up- or down-regulated). The *y*-axis shows the –log₁₀ *P*-value, representing the statistical significance of the change. Proteins that are significantly upregulated appear in the upper right quadrant, while those significantly downregulated appear in the upper left quadrant. B) Swimming motility test of *E. asburiae* AG129 wild type and AG129*easI* mutant and complemented strain AG129*easI*(pBBRMCS1*easI*) on TS solid medium (3% agar). To assess statistical significance, One-way ANOVA and Dunnet multiple comparison test was used. All data are presented as means ± standard error (SD). The error bars indicate standard deviations. C) β-galactosidase assays showing the promoter activity in AG129(pMP220*hyp1*), AG129*easI*(pMP220*hyp1*), and chemically complemented AG129*easI*(pMP220*hyp1*) with 1 μM C4-AHL, measured at 7 and 16 hr of growth, respectively. D) β-galactosidase assays showing the promoter activity in AG129(pMP220*hcp*), AG129*easI*(pMP220*hcp*), and chemically complemented AG129*easI*(pMP220*hcp*) with 1 μM C4-AHL, measured at 7 and 16 hr of growth, respectively. E) β-galactosidase assays showing the promoter activity in AG129(pMP220*hyp6*), AG129*easI*(pMP220*hyp6*), and chemically complemented AG129*easI*(pMP220*hyp6*) with 1 μM C4-AHL, measured at 7 and 16 hr of growth, respectively. The graphs present means ± standard error of the mean (SD) with statistical significance determined using a t-test, analyzed with GraphPad Prism 9.2 (GraphPad Software, Inc.). Significance levels are indicated as *P < 0.05, **P < 0.005, ***P < 0.0005, and ****P < 0.00005.

Secretome analysis revealed 67 proteins upregulated in the WT compared to the *easI* mutant, and 69 proteins upregulated in the *easI* mutant relative to the WT, based on log_2_ fold-change values (Fig. 3A, Table S6). Several of the proteins most strongly expressed in the WT were among the most highly downregulated in the *easI* mutant (fold change –2 to –8) (Table S6, protein IDs in red), indicating a strong dependency on AHL signaling. Strikingly, five of these belonged to the Type VI secretion system (T6SS), including VgrG, Hcp, an RHS-repeat protein, and two hypothetical proteins located within the same T6SS gene cluster.

Additionally, other highly expressed proteins in the wild type strain included: a Cu-Zn family superoxide dismutase, a filamentous hemagglutinin, an adhesin HecA-like repeat protein and three hypothetical proteins. Conversely, the mutant strain displayed elevated levels of several flagella-associated proteins, pointing toward a possible shift in motility-related behavior in the absence of EasI-mediated signaling (Table S6).

Together, the T6SS components were among the clearest and most biologically meaningful QS-regulated targets, providing a direct rationale for selecting these genes for further validation.

### The AHL QS system regulates T6SS and swimming motility in *E. asburiae* AG129

As the secretome analysis revealed that the levels of T6SS-associated proteins were regulated by AHL QS it was of interest to further validate this result. The promoter regions of three genes, which are part of the T6SS cluster (*hyp1, hcp*, and *hyp6*) (Fig. S1) were cloned in the pMP220 promoter probe vector which carries the β-galactosidase reporter gene as described in the Materials and methods section. Promoter activity of three genes was assessed in *E. asburiae* AG129 WT and the *easI* mutant using the following genetic backgrounds: WT strains AG129(pMP220*hyp1*), AG129(pMP220*hcp*), AG129(pMP220*hyp6*), and the mutant counterparts AG129*easI*(pMP220*hyp1*), AG129*easI*(pMP220*hcp*), and AG129*easI*(pMP220*hyp6*). All three promoters showed statistically significant higher activity in the WT compared to the *easI* mutant. Chemical complementation of the mutant with C4-AHL restored promoter activity to WT levels. The *hyp1* promoter showed strong activity at both 7 and 16 hr (Fig. 3C), while *hcp* and *hyp6* were also QS-regulated but with lower activity (Figs 3D–E). In summary, T6SS expression in *E. asburiae* AG129 is regulated either directly or indirectly by AHL QS. This aligns with and supports the secretome analysis results.

In addition, secretome analysis revealed elevated levels of several flagella-associated proteins in the *easI* mutant, prompting us to examine whether this translated into altered motility. We therefore performed an *in vitro* swimming assay (Fig. 3B) comparing AG129 wild type, the *easI* mutant, and the complemented strain. The wild type showed no detectable swimming activity, whereas disruption of the AHL synthase gene restored clear swimming motility. These findings corroborate the secretome results and the rhizosphere/endosphere colonization patterns, reinforcing that the mutant exhibits enhanced motility relative to the wild type, and that this behavior is modulated by the AHL QS system.

### *Enterobacter asburiae* AG129 T6SS contact-dependent killing assays

A bacterial killing assay was conducted to investigate the role of AHL QS in T6SS-mediated contact-dependent killing, using *E. coli* as target strain. Co-spotting experiments on solid media showed a minimal but significant reduction in *E. coli* CFUs when co-cultured with the attacker AG129 WT compared to the AG129*easI* mutant or the complemented strain, both after 4 and 18 hr of incubation (Fig. S3). In contrast, liquid co-culture assays revealed no significant changes in target *E. coli* CFUs when co-cultured with either the wild-type or mutant and complemented strains (Fig. S3). These results supported the finding that *E. asburiae* AG129 displays a QS-regulated, contact-dependent killing phenotype specifically on solid surfaces, likely mediated by the T6SS.

### The AHL-QS system regulates the production of several bacterial volatile compounds (VOCs) in *E. asburiae* AG129

**Figure 4.**
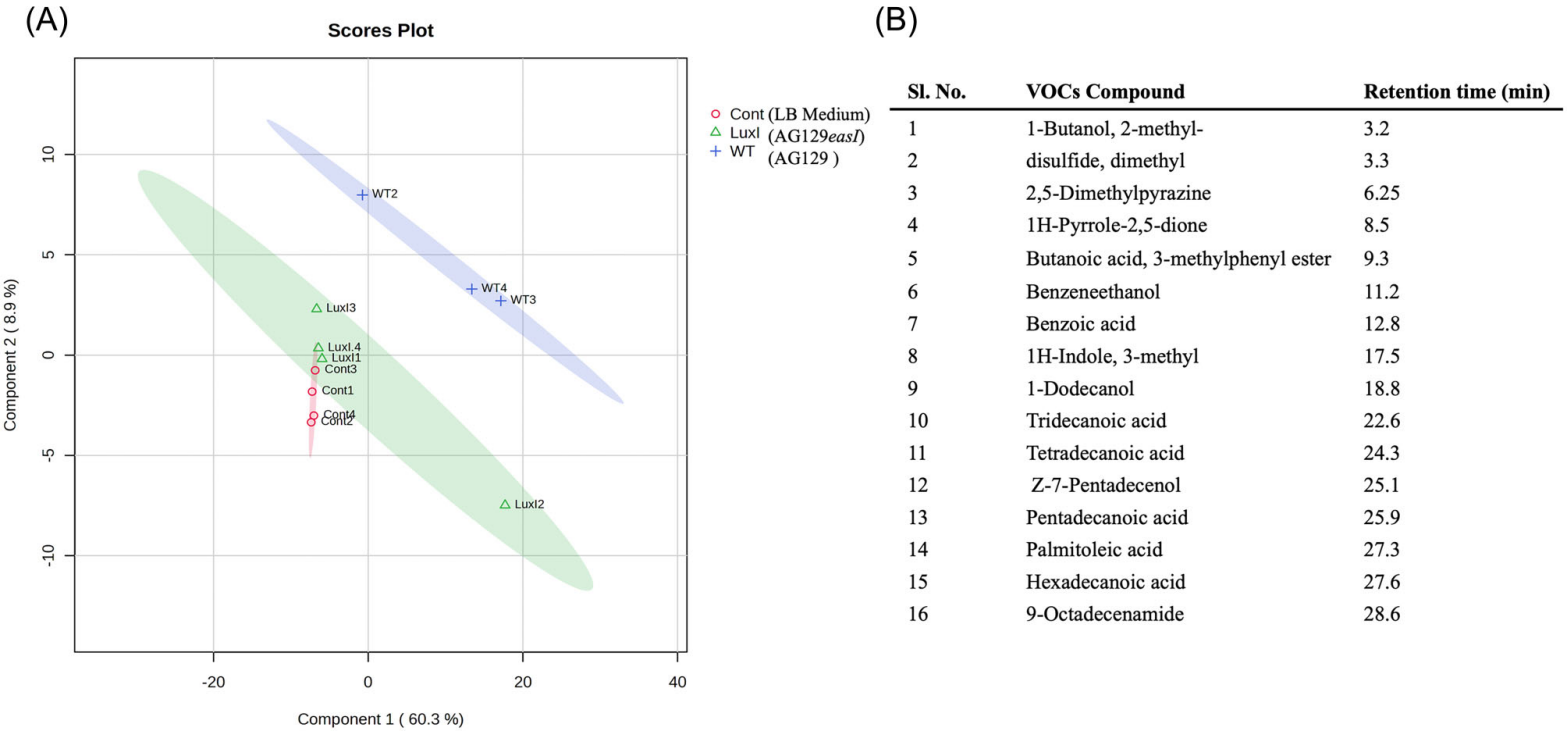
AHL QS system regulates VOCs production in *E. asburiae* AG129. A) The AG129 wild type emits significantly higher levels of volatile compounds compared to the AG129*easI* mutant, demonstrating that the concentration of these compounds is greater in the wild type than in the mutant and not detected at all in the control medium. B) Main Volatile Compounds significantly more abundantly emitted in the wild type compared to the *easI* mutant.

Volatile compounds (VOCs) produced by bacteria often play a role in microbial ecology, influencing interactions among microorganisms and with plants (Venturi and Keel 2016: 187–98). It was of interest to determine the VOCs produced by *E. asburiae* AG129 and their possible regulation by AHL QS. The VOCs produced by AG129 WT and AG129*easI* mutant strains were trapped and determined as described in the Materials and Methods section. Gas chromatography–mass spectrometry analysis revealed that several volatile compounds were emitted in significantly higher abundance in the wild type strain compared to the mutant and control medium (Fig. 4A). The identified VOCs belong to different chemical categories, including alcohols (phenolic), disulfides, pyrazines, pyrroles, esters, acids, and indoles. A total of 16 VOCs were emitted abundantly by the wild-type strain, but their production was notably significantly reduced or absent in the *easI* mutant, suggesting that these VOCs could be regulated by AHL QS. The 16 VOCs emitted in higher abundance by the wild-type strain (Fig. 4B) included butanol (2-methyl), dimethyl disulfide, 2,5-dimethylpyrazine, 1H-pyrrole-2,5-dione, 3-methylphenyl butanoic acid ester, benzene ethanol, benzoic acid, 3-methyl-1H-indole, 1-dodecanol, tridecanoic acid, tetradecanoic acid, Z-7-pentadecenol, pentadecanoic acid, palmitoleic acid, hexadecanoic acid, and 9-octadecenamide.

To evaluate whether VOCs emitted by AG129 WT, and the *easI* mutant exhibited different antimicrobial activity, we performed double-compartment assays using the bacterial pathogen *Dickeya zeae* and the fungal pathogen *Botrytis sp*. as targets. VOCs produced by the *easI* mutant caused an inhibition of *D. zeae*, as shown by the reduced growth area (Fig. S4A) and significantly lower CFU counts compared with both the wild type and complemented strain (Fig. S4B). A similar pattern was observed against *Botrytis sp*., with the *easI* mutant displaying inhibitory effect on fungal growth (Fig. S4C). Together, these results suggest that the EasI/R AHL-QS system in *E. asburiae* AG129 is involved in the regulation of some VOCs having antagonism activity toward both bacterial and fungal pathogens.

## Discussion

This study characterized the AHL QS system of the rice endophyte *Enterobacter asburiae* AG129. The strain possesses one AHL QS system, EasI/R, which produces and responds to *N*-butanoyl homoserine lactone (C4-AHL). Although previous work on related species *(E. cloacae, E. sakazakii*, and other *Enterobacter* isolates) has reported the presence of orthologous LuxI/LuxR-type systems, the available studies remain fragmented, strain-specific, and often lack rigorous functional validation. As a result, the ecological and physiological roles of AHL-mediated QS in the genus remain poorly defined. By providing a controlled, mechanistic characterization of the EasI/R system in AG129, our work contributes to the molecular and ecological relevance of QS-regulated traits in the *Enterobacter* species. (Andrés-Barrao et al. 2017: 2023, Dupont et al. 2023: 1 264 801, Lau et al. 2018: e00610).

Whole-genome sequencing revealed that the *easI* and *easR* genes are positioned adjacent to each other and oriented in opposite directions. This gene arrangement, as well as the genetic context next to the QS system, is conserved among various *Enterobacter* species and related genera, such as *Lelliottia* and *Leclercia*, all within the Enterobacteriaceae family. The wild-type strain established stable colonization in both the rhizosphere and endosphere, whereas the *easI* mutant exhibited statistically significant higher colonization levels. This may be due to differential regulation of secreted proteins or negative regulation by the AHL QS system, which could repress a phenotype that enhances root colonization. Alternatively, the QS mutant might possess a metabolic advantage, allowing faster growth under certain conditions within plant roots (Synek et al. 2021: 6223–40, Taghavi et al. 2010: e1000943). QS positively regulates the expression of several secreted proteins, including the adhesin filamentous hemagglutinin (FHA), HecA-like repeat proteins, and the Cu-Zn superoxide dismutase (SOD). FHA and HecA proteins, essential for bacterial adhesion, facilitate host attachment and immune evasion through a two-partner secretion system, a strategy used by pathogens like *Pseudomonas aeruginosa* and *Ralstonia solanacearum* to support biofilm formation and persistence (Ossowicki et al. 2017: e0174362, Popova et al. 2014: 125 704, Quebatte et al. 2010: 3352–67, Rani et al. 2023: 108 078, Rojas et al. 2002: 13142–7). In contrast, flagellar-like proteins were upregulated in the *easI* mutant, indicating that QS exerts a negative regulatory effect (Chagnot et al. 2013: 303, Molina et al. 2006: 639–47). This finding aligns with the increased root colonization observed in the *easI* mutant and suggests that the AHL-QS system in *Enterobacter* plays a role in regulating bacterial motility. Interestingly, no differences in motility were detected between the wild type and mutant strains under *in vitro* conditions, which differ significantly from the plant-associated environment. However, previous studies have indicated that mutations in flagellar-like proteins and QS genes can enhance root colonization, although the mechanisms by which the loss of motility may facilitate this process remain unclear (Wang et al. 2023: e04346-22). In *E. cloacae*, the inactivation of the QS-related transcriptional regulator *sdiA* increases root colonization and biofilm formation, thus also having a negative impact of QS on cellular adhesion (Dos Reis et al. 2019: 30–7). Interestingly, upregulation of flagellar proteins in the *easI* mutant aligns with studies showing that flagellar components, which contribute to motility, also play roles in adhesion and initial host surface attachment (Kim et al. 2019: 329–34). The increased flagellar protein expression in QS mutants may compensate for the altered regulatory balance, promoting colonization despite reduced adhesion protein levels. QS negatively regulates root colonization by modulating the interplay between motility and adhesion, with motility aiding initial root colonization and adhesion ensuring a stable presence (Makhubu et al. 2023: 729–35).

The secretome study demonstrated that the *easI/R*-mediated QS system regulated the T6SS in *E. asburiae* AG129. Five proteins in the T6SS cluster, namely Valine–glycine repeat protein G (VgrG), Hemolysin-coregulated protein (Hcp), Rearrangement hotspot repeat-associated protein (RHS), and two hypothetical proteins had significant log2 fold level changes ranging from −2 to −8 in the *easI* mutant compared to the wild-type. In addition, transcriptional regulation of key T6SS genes (*hyp1, hcp*, and *hyp6*) was positively influenced by AHL-mediated QS. The T6SS, a contractile apparatus used to inject toxic effectors into competing cells, plays roles in virulence, biofilm formation, interbacterial competition and contact-dependent inhibition (MacIntyre et al. 2010: 19520–4, Trunk et al. 2018: 920–31). QS has been previously reported to regulate T6SS activation, possibly linking population density to contact-dependent killing, as observed in *Vibrio cholerae* and *Vibrio fluvialis* (Pandey and Banerjee 2015: 330–7). T6SS is more effective on solid surfaces, where direct cell contact occurs, than in liquid cultures. This QS-T6SS interplay likely influences microbial interactions in the root microbiome, indicating the ecological significance of QS in endophytic bacteria.

Bacterial VOCs play crucial roles in ecological interactions among bacteria, influencing competition, plant growth, and community dynamics (Dupont et al. 2023: 1 264 801, Garbeva et al. 2014: 289). *Enterobacter* spp., have been recently shown to produce VOCs such as acetoin and 2,3-butanediol, which enhance plant growth (Andrés-Barrao et al. 2017: 2023, Synek et al. 2021: 6223–40). The production of VOCs is regulated by various environmental factors, including nutrient availability (Taghavi et al. 2010: e1000943). However, the specific role of QS in regulating VOC production in *Enterobacter* and other bacterial species remains unknown. This study indicated that a diverse array of VOCs was regulated by AHL QS in strain AG129; 16 VOCs were emitted in higher abundance in the AG129 WT compared to the mutant AG129*easI*. Certain VOCs have been shown to affect plant growth and development (Chagnot et al. 2013: 303, Molina et al. 2006: 639–47, Quebatte et al. 2010: 3352–67, Rojas et al. 2002: 13142–7). The ability of *E. asburiae* AG129 to produce VOCs in response to AHL signaling may enhance its competitiveness in the root environment, where volatile emissions can deter pathogens or attract beneficial organisms (Ossowicki et al. 2017: e0174362). VOCs can also serve as long-distance chemical cues within microbial communities, facilitating communication and interaction among species. The VOCs identified in this study, such as 1-butanol, disulfide dimethyl, and 2,5-dimethylpyrazine, are associated with various biological activities, including promoting plant growth and exhibiting biocontrol activity, and antimicrobial properties (Popova et al. 2014: 125 704, Rani et al. 2023: 108 078, Wang et al. 2023: e04346-22). Additionally, VOCs like 9-octadecenamide have demonstrated antibacterial properties, which may help the producing bacteria suppress competitors in their surroundings. This compound belongs to a broader category of fatty acid amides linked to various biological activities, including antimicrobial effects (Dos Reis et al. 2019: 30–7, Fan et al. 2024: 130 525, Kim et al. 2019: 329–34, Makhubu et al. 2023: 729–35). Moreover, previous studies demonstrated that benzeneethanol, a phenylethyl alcohol produced by *Daldinia* and *Phomopsis* spp. displays antimicrobial activity and is a potential signaling molecule in plant growth promotion (Pandey and Banerjee 2015: 330–7, Singh et al. 2011: 729–39). Furthermore, hexadecanoic acid, produced by *Bacillus* and *Pseudomonas* spp., is recognized for its antifungal properties and ability to promote plant growth (Dos Reis et al. 2019: 30–7, Singh et al. 2011: 729–39). These findings suggest that the VOCs produced by *E. asburiae* AG129 and regulated by AHL-QS could enhance its ecological fitness by providing a competitive advantage against pathogens in the root environment. However, future *in vivo* experiments under plant-associated conditions will be essential to determine whether QS directly contributes to plant growth promotion and to clarify its functional role in plant–microbe interactions.

In summary, this study sheds light on the AHL QS system in *E. asburiae* AG129, and for the first time in bacterial root endophytes. The AHL QS system was found to positively regulate the T6SS and the production of various VOCs, both of which likely contribute to bacteria–bacteria and plant–bacteria interactions. On the other hand, QS negatively affected root colonization and swimming motility. These findings highlight the role of AHL QS in microbial ecology and in association with the plant host. Future research could explore the specific roles of the T6SS and VOCs in plant–bacteria interactions and the molecular mechanisms governing their regulation by the AHL QS system.

## Supplementary Material

fiaf120_Supplemental_Files

## Data Availability

All the data published is included in the manuscript or supplementary material.
