# Supplementary material for: AHL quorum sensing regulates T6SS and volatiles production in rice root-colonizing Enterobacter asburiae AG129
Source: FEMS Microbiol Ecol. 2025 Dec 8;102(1):fiaf120. doi: 10.1093/femsec/fiaf120 (PMC12728822; doi:10.1093/femsec/fiaf120)

FragPipe-Analyst report

19 February, 2024

Method details

The raw data files were analyzed using FragPipe to obtain protein identifications and their respective label-free quantification values using in-house standard parameters. Statistical analysis was performed based on the combined_protein.tsv file. First, contaminant proteins were filtered out. In addition, proteins that were not identified/quantified consistantly in same condition have been removed as well. The MaxLFQ intensity values were converted to log2 scale, samples were grouped by conditions and missing values were imputed using the 'Missing not At Random' (MNAR) method, which uses random draws from a left-shifted Gaussian distribution of 1.8 StDev (standard deviation) apart with a width of 0.3. Protein-wise linear models combined with empirical Bayes statistics were used for the differential expression analyses. The *limma* package from R Bioconductor was used to generate a list of differentially expressed proteins for each pair-wise comparison. A cutoff of the *adjusted p-value* of 0.05 (Benjamini-Hochberg method) along with a |log2 fold change| of 1 has been applied to determine significantly regulated proteins in each pairwise comparison.

Quick summary of parameters used:

- Tested pairwise comparisons = delta_vs_wt
- Adjusted *p-value* cutoff <= 0.05
- Log fold change cutoff >= 1

Results

FragPipe result output contains proteins groups of which *144* proteins were reproducibly quantified.

38 proteins differ significantly between samples.

Exploratory Analysis (QC Plots)

Principle Component Analysis (PCA) plot

```
## Warning: Use of `pca_df[[indicate[1]]]` is discouraged. Use
## `.data[[indicate[1]]]` instead.
```

```
## Warning: Use of `pca_df[[indicate[2]]]` is discouraged. Use
## `.data[[indicate[2]]]` instead.
```

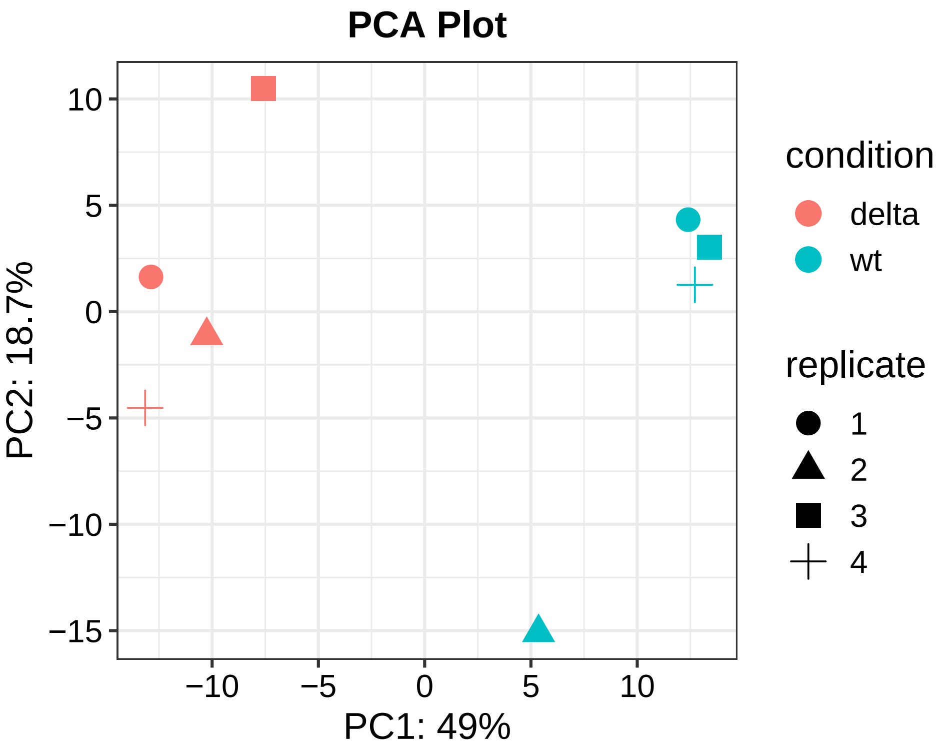

Sample Correlation matrix

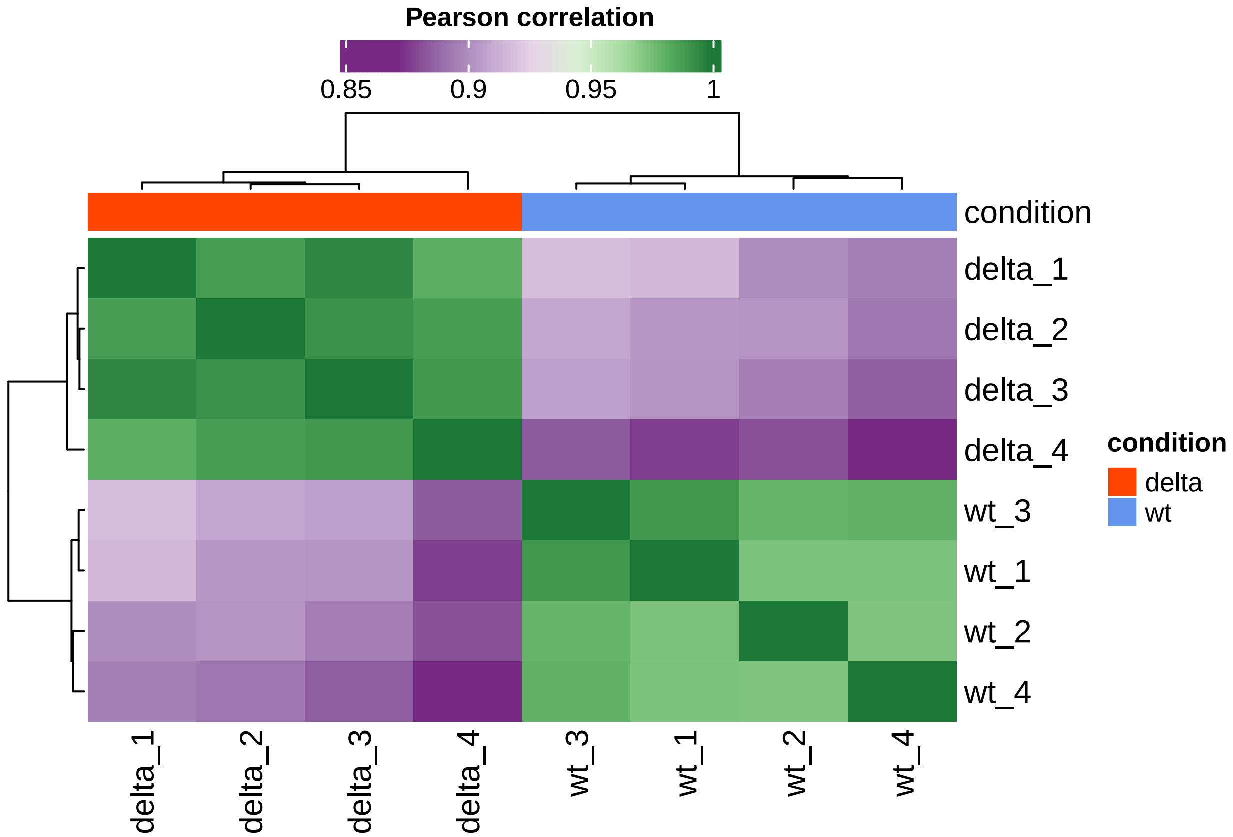

Sample Coefficient of variation (CVs)

```
## `summarise()` has grouped output by 'rowname'. You can override using the
## `.groups` argument.
```

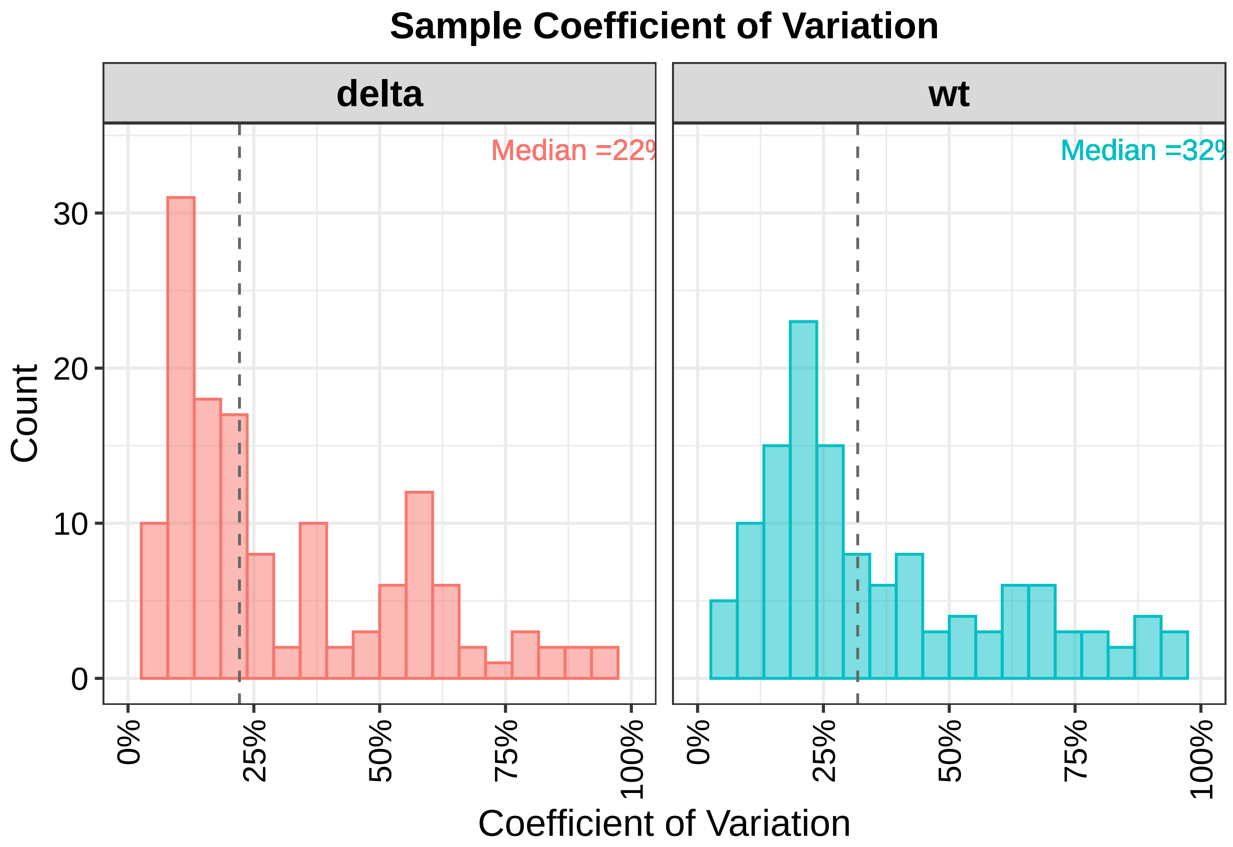

Proteomics Experiment Summary

Protein quantified per sample (after pre-processing).

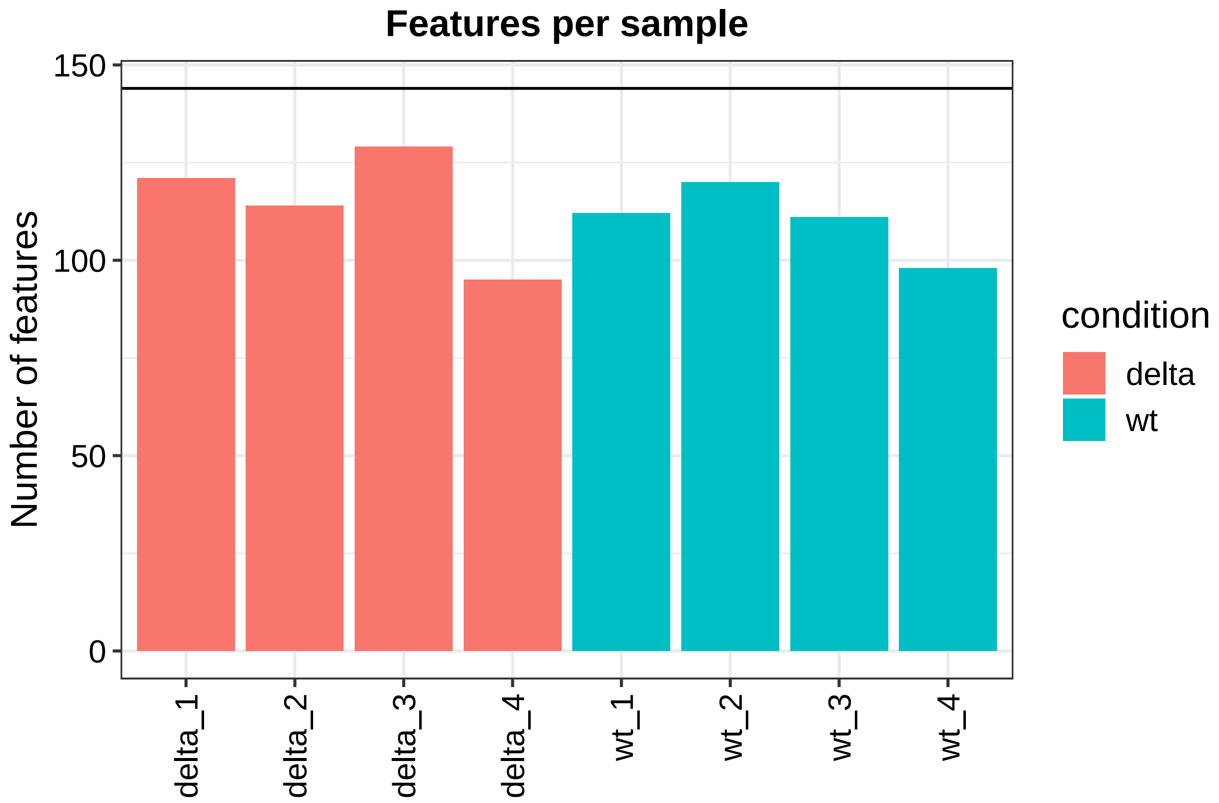

Protein overlap in all samples.

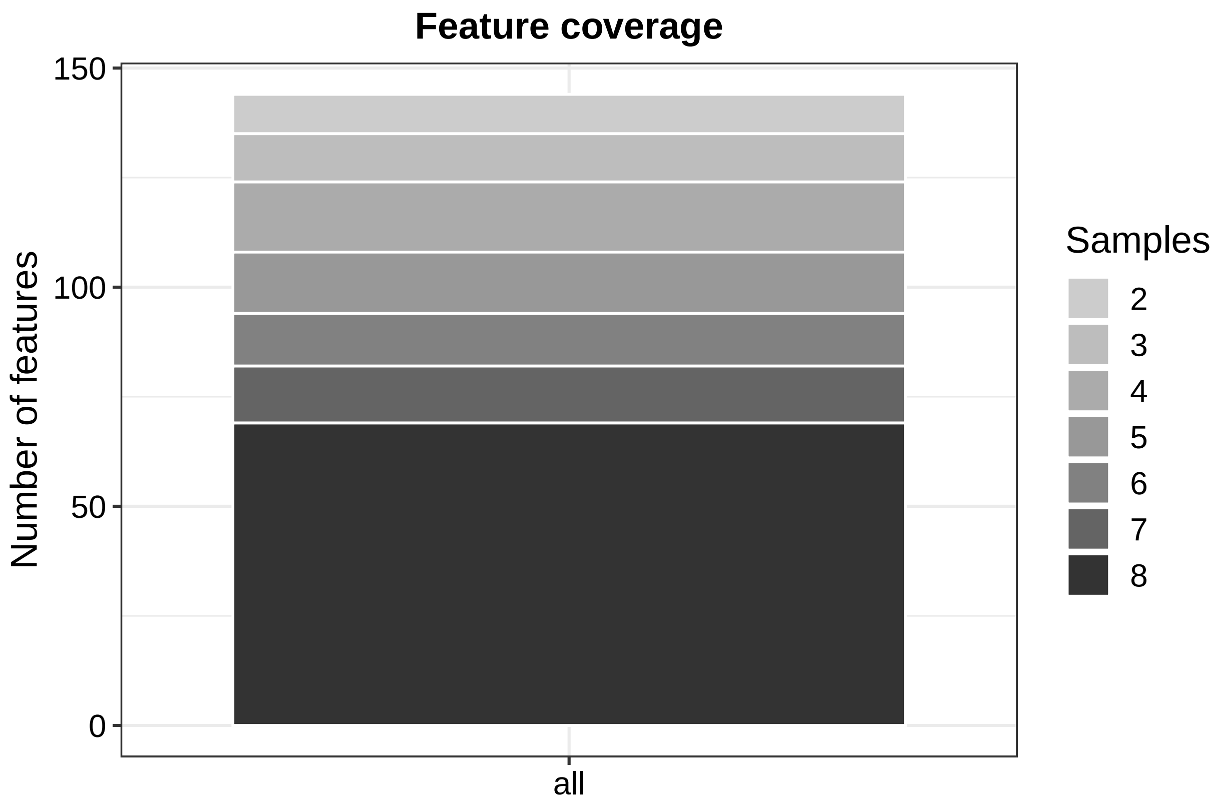

Missing Value handling

Missing value heatmap

A heatmap for proteins with missing value in each dataset. Each row represent a protein with missing value in one or more replicate. Each replicate is clustered based on presence of missing values in the sample.

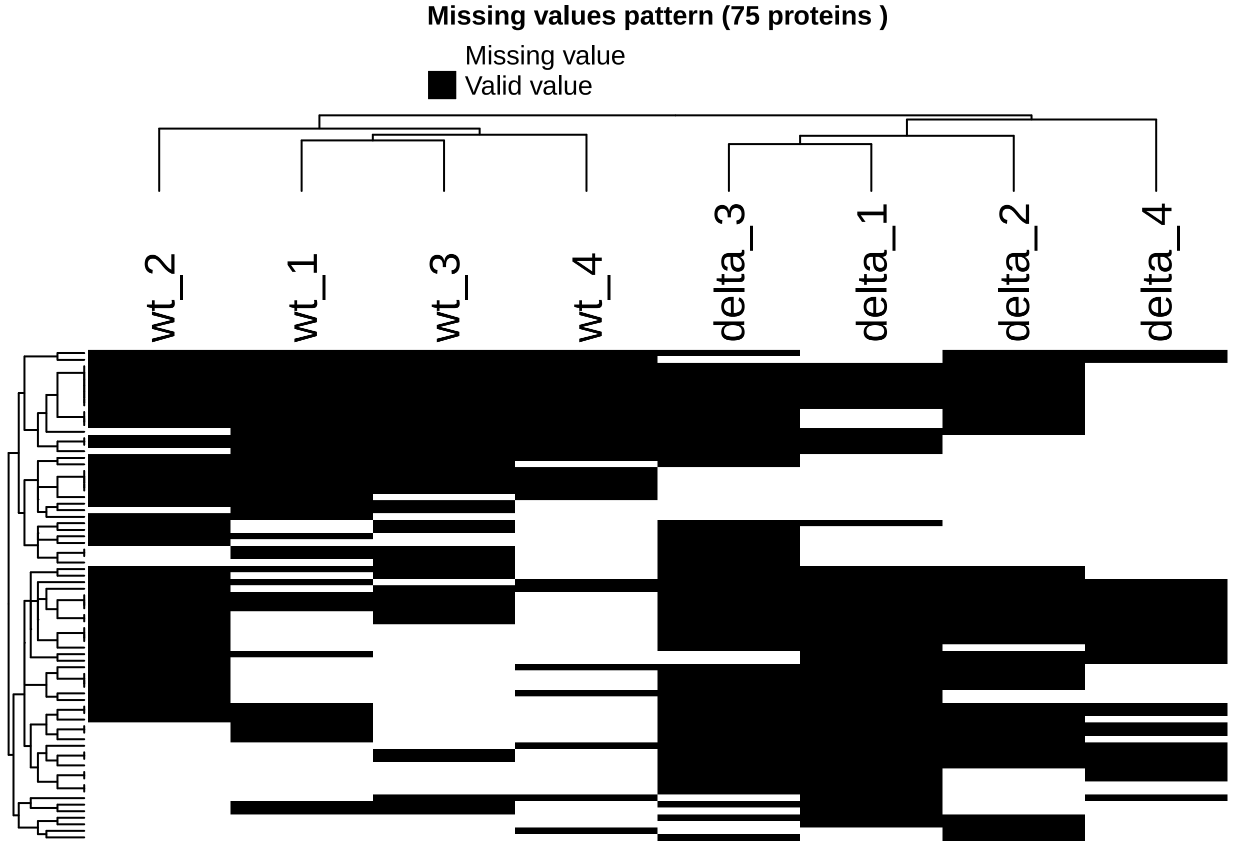

Missing value distribution

Protein expression distribution before and after imputation. The plot showing the effect of imputation on protein expression distribution.

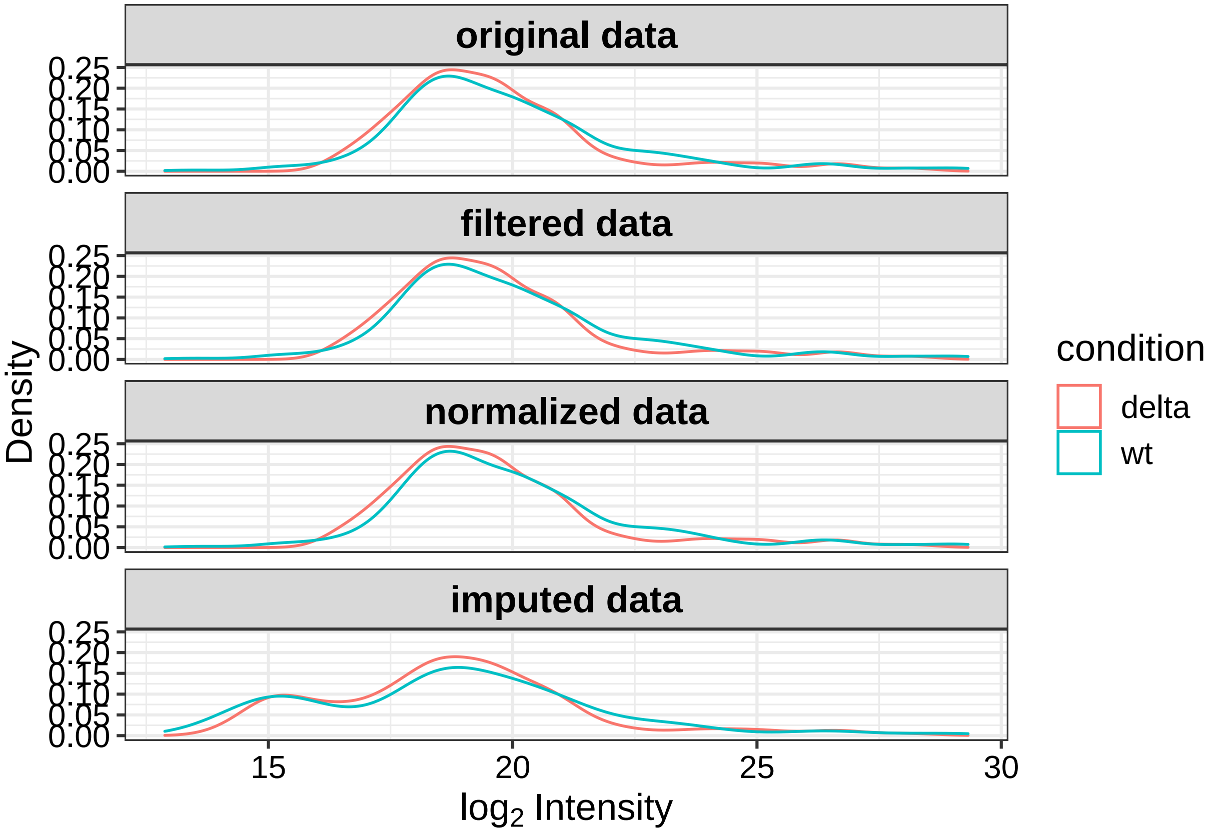

Differential Expression Analysis (Results Plots)

Heatmap

A plot representing an overview of expression of all significant (differencially expressed) proteins (rows) in all samples (columns).

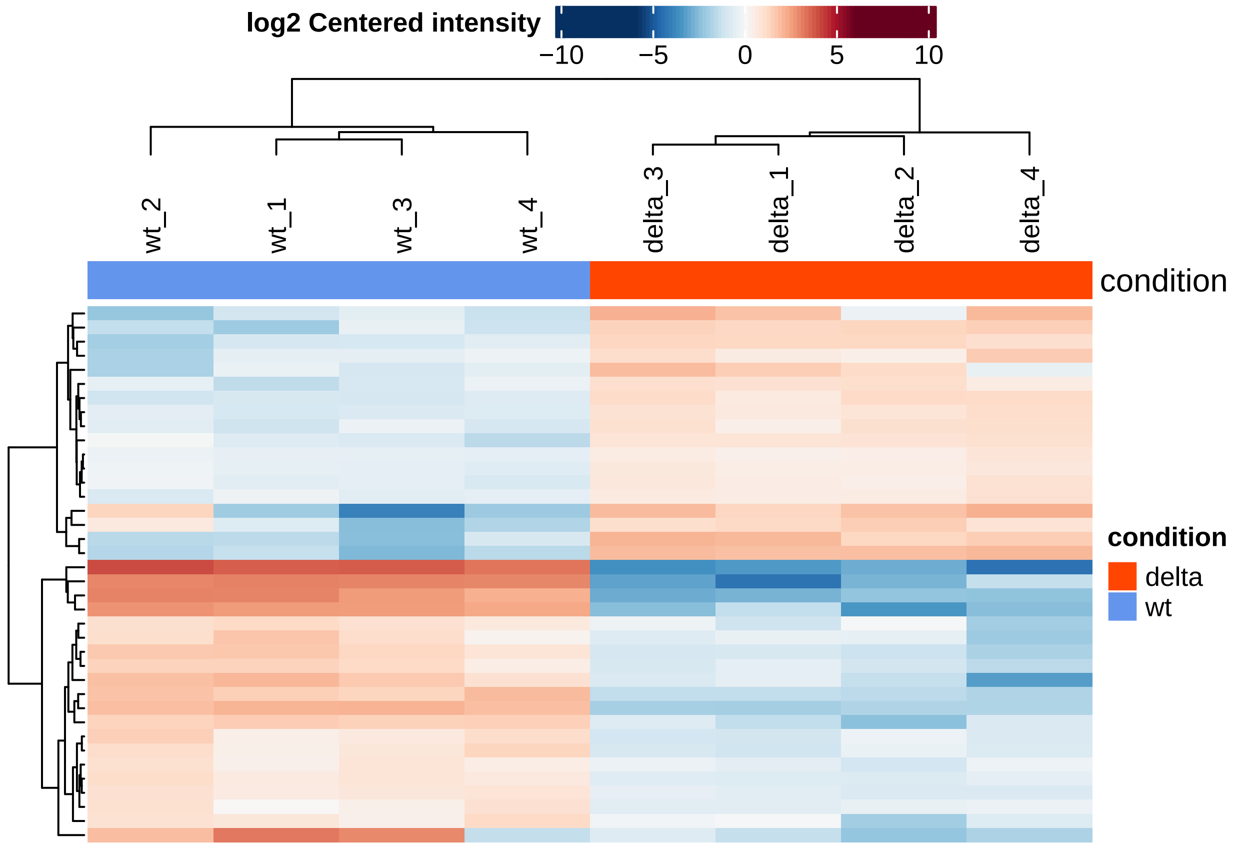

Volcano Plots

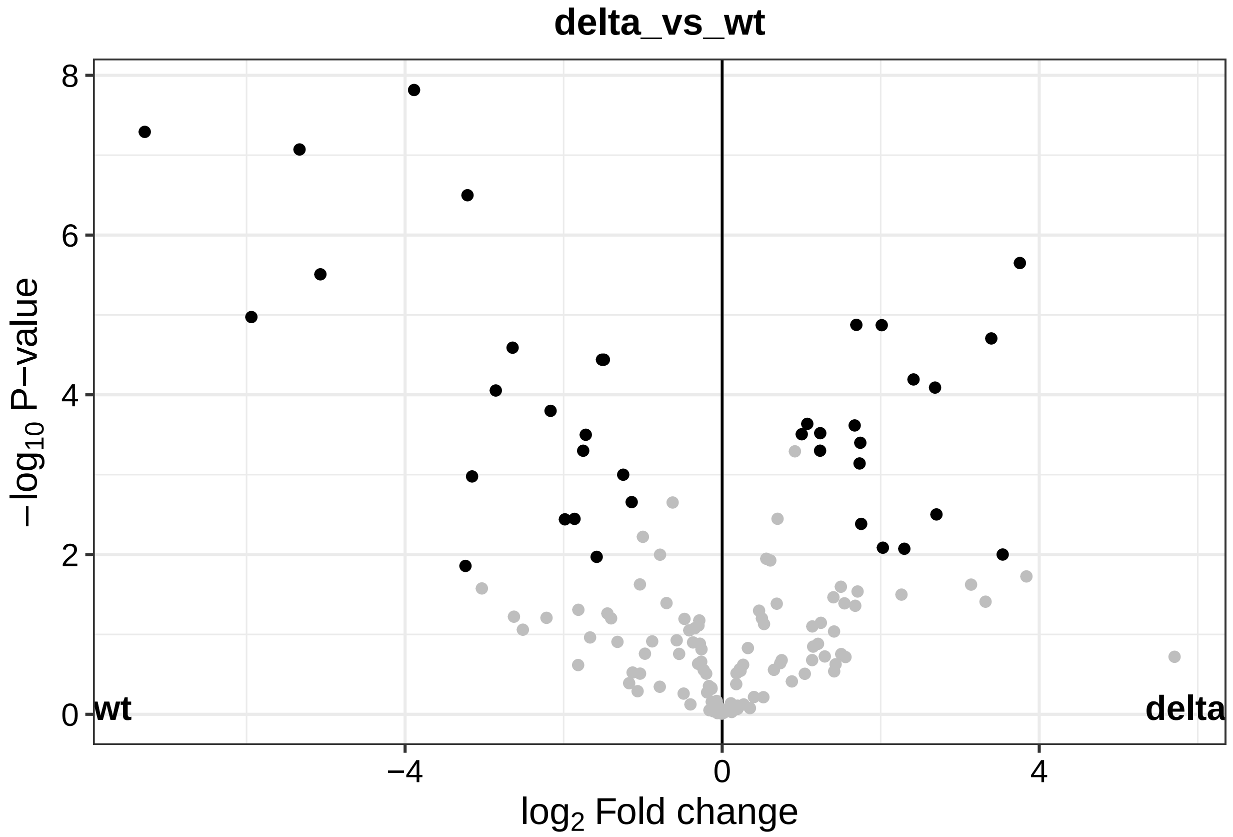

Supplement: fiaf120_Supplemental_Files [file fiaf120_supplemental_files.zip › FragPipe-Analyst_report-chandan.pdf]
