# Supplementary material for: AHL quorum sensing regulates T6SS and volatiles production in rice root-colonizing Enterobacter asburiae AG129
Source: FEMS Microbiol Ecol. 2025 Dec 8;102(1):fiaf120. doi: 10.1093/femsec/fiaf120 (PMC12728822; doi:10.1093/femsec/fiaf120)
Supplement: fiaf120_Supplemental_Files [file fiaf120_supplemental_files.zip › supplementary material-EnterobacterF_25.04.25_REV.docx]

***N*-acyl homoserine lactone quorum sensing in root associated *Enterobacter asburiae* AG129**

Chandan Kumar^a^, Iris Bertani^a^, Paolina Garbeva^c^, Michael Myers^a^, Cristina Bez^a^, Vittorio Venturi^a,b*^

1. International Center for Genetic Engineering and Biotechnology, Trieste, Italy
2. African Genome Center, University Mohammed VI Polytechnic (UM6P), Ben Guerir, Morocco
3. Department of Microbial Ecology, The Netherlands Institute of Ecology (NIOO-KNAW), Droevendaalsesteeg 10, 6708 PB Wageningen, the Netherlands

* Corresponding: Vittorio Venturi: vittorio.venturi@um6p.ma; venturi@icgeb.org

**Supplementary figure**

**SF 1:** Proteins within the T6SS apparatus including the VgrG, Hcp, RHS repeat-associated protein, and two hypothetical proteins. The number corresponds to the gene.


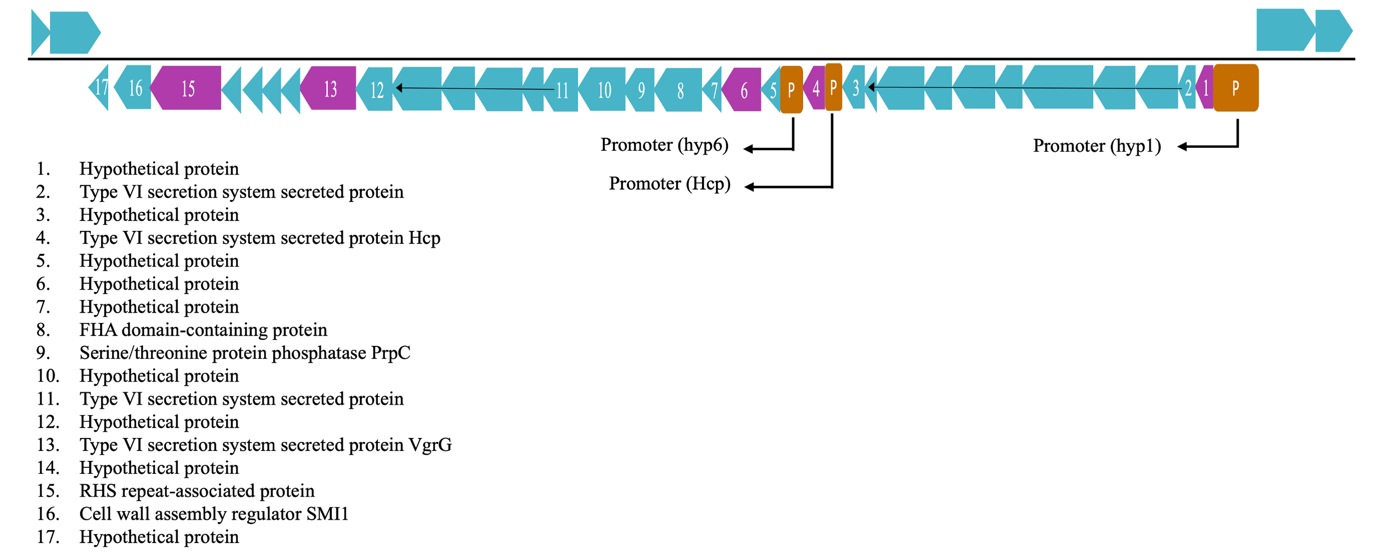


**SF 2:** AHL production was assessed using cross-streak assays with the *Chromobacterium* *violaceum* CV026 biosensor strain, in which the appearance of purple pigment indicates the presence of AHL signals.

**
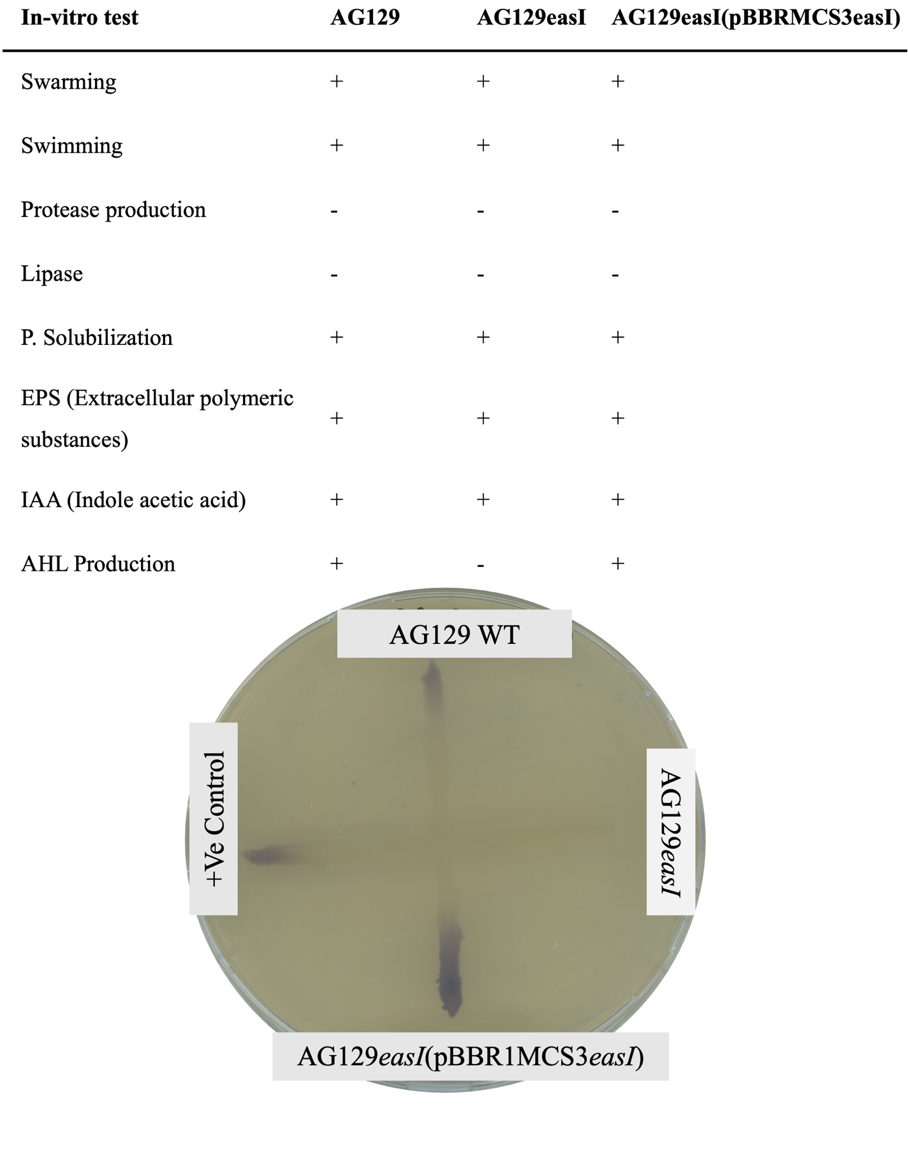
**

**Supplementary table**

**ST 1**: Plasmid and strain used in this study

| **Plasmid/Strain** | **Relevant characteristic** | **Reference** |
| --- | --- | --- |
| **Plasmids** | | |
| pGEM-T easy | Cloning vector; AmpR LacZ reporter | Promega |
| pLAFR3-Tc | Broad-host-range cosmid cloning vector, IncP1; TetR | (Staskawicz, Dahlbeck, Keen, & Napoli, 1987) |
| pPH1JI-Gm | IncP1, Gm^r^ | (Hirsch & Beringer, 1984) |
| pBBR1MCS-3-Tc | Broad-host-range suttle cloning vector | (Kovach et al., 1995) |
| pMP220-Tc | Promoter probe vector, IncQ; TetR | (Spaink, Okker, Wijffelman, Pees, & Lugtenberg, 1987) |
| pRK2013-Km | Tra^+^Mob^+^ colE1 replicon Km^R^ | (Figurski & Helinski, 1979) |
| pUCK4 | Plasmid encoding a Kan^r^ gene cassette | Pharmacia Biotech |
| pGEM-T*easI*::Kan | Kan^r^ gene of pUCK4 inserted into the unique pstI site | This study |
| pLAFR3*easI*::kan | pLAFR3 carrying *easI*::Kan | This study |
| pBBR1MCS-3*easI* | pBBR1MCS-3 carrying *easI* gene with flanking region (200 nucleotides) from both the site | This study |
| pUC57(hyp1) | 1101bp promoter fragment XbaI and KpnI restriction sites at the 5’ and 3’ end respectively | This study |
| pUC57(Hcp) | 287bp promoter fragment XbaI and KpnI restriction sites at the 5’ and 3’ end respectively | This study |
| pUC57(hyp6) | 694bp promoter fragment PstI and KpnI restriction sites at the 5’ and 3’ end respectively | This study |
| pMP220(hyp1) | Promoter probe constructs pMP220 carrying hyp1 promoter | This study |
| pMP220(Hcp) | Promoter probe constructs pMP220 carrying Hcp promoter | This study |
| pMP220(hyp6) | Promoter probe constructs pMP220 carrying hyp6 promoter | This study |
| **Bacterial strain** | | |
| CV026 | Violacein pigment | (McClean et al., 1997) |
| DH5α | *Escherichia coli* | Lab collection |
| AG129 | *Enterobacter asburiae* wild type strain | (Bertani et al., 2016) |
| AG129Rif^R^ | AG129 derivatives, rifampicin (Rif) resistance (R) | This study |
| AG129easI | *AG129easI* knowckout mutant, km^R^ | This study |
| AG129easI(pBBR1MCS-3*easI*) | *AG129easI* mutant carrying the *easI* gene in pBBR1MCS-3 | This study |
| AG129(pBBR1MCS-3) | AG129 wild type carrying pBBR1MCS-3 vector | This study |
| AG129easI(pBBR1MCS-3) | *easI* knowck out mutant caring pBBR1MCS-3 empty vector | This study |
| AG129(pMP220hyp1) | AG129 WT carrying Promoter probe constructs caring pMP20hyp1 promoter | This study |
| AG129(pMP220 Hcp) | AG129 WT carrying Promoter probe constructs pMP20Hcp promoter | This study |
| AG129(pMP220hyp6) | AG129 WT carrying Promoter probe constructs pMP20hyp6 promoter | This study |
| AG129*easI*(pMP220hyp1), | *easI* knowck out mutant carrying Promoter probe constructs pMP20hyp1 promoter | This study |
| AG129*easI*(pMP220Hcp), | *easI* knowck out mutant carrying Promoter probe constructs pMP20Hcp promoter | This study |
| AG129*easI*(pMP220hyp6), | *easI* knowck out mutant carrying Promoter probe constructs pMP20hyp6 promoter | This study |
| AG129(pMP220) | *Enterobacter asburiae* wild type carrying pMP20 vector | This study |
| AG129esaI(pMP220) | *easI* knowck out mutant carrying pMP20 vector | This study |

**ST 2:** List of primers used in this study

| **Primer Name** | **Sequence (5’ to 3’)** | **Description** | **Reference/**  **Source** |
| --- | --- | --- | --- |
| luxIBfw | cgc**ggatcc**gcgTGCAAGAGTAGCACAGCATTAAG | For *easI* mutant contruction underline is restriction enzyme | This study |
| luxIpRw2 | GAATAACTTCAACTTTCCAGTTctgcagatAGCAAGTATGTCGTGTTTGG | For *easI* mutant contruction underline is restriction enzyme | This study |
| luxIPfw3 | ATctgcagAACTGGAAAGTTGAAGTTATTCAACGCGGTGTTTCTGAAAAAGGG | For *easI* mutant contruction underline is restriction enzyme | This study |
| luxIBRw | cgcggatccGCGGTGATGTCATTCTGTCGGATGG | For *easI* mutant contruction underline is restriction enzyme | This study |
| LuxIcomFK | atggtacCTTCTTACGCTCTGTACCCGAAAC | For *easI* complemetation contruction underline is restriction enzyme | This study |
| LuxIcompRX | gctctagagcCGTGAGCGACAAATCCTTAAATGG | For *easI* complemetation contruction underline is restriction enzyme | This study |
| Kan ext FP | TGGAATTTAATCGCGGCCTCG | Amplification of Km cassette | This study |
| Kan ext RP | CATCTTCCCGACAACGCAGAC | Amplification of Km cassette | This study |
| pMP220 forward primer | GCTGAACGGTCTGGTTA | Verify promoter probe construction | Universal primer (Lab) |
| pMP220 reverse primer | CCAAAAATGGCAGCCAAG | Verify promoter probe construction | Universal primer (Lab) |

**ST 3a:** The plant growth-promoting (PGP) traits of *E. asburiae* strain AG129 are indicated as follows: (+) denotes the presence of activity, while (−) denotes the absence of activity.

| **Plant-associated phenotypes (*in vitro*)** | **AG129 WT** | **AG129*easI*** | **AG129*easI* (pBBRMCS1*easI*)** |
| --- | --- | --- | --- |
| **Proteolytic activity** | **-** | **-** | **-** |
| **Lipolytic activity** | **-** | **-** | **-** |
| **P. solubilization** | **+** | **+** | **+** |
| **EPS production** | **+** | **+** | **+** |
| **IAA production** | **+** | **+** | **+** |

**ST 4:** Summary of the functional annotation of the *Enterobacter asburiae AG129*

| Features | Genome Statistic |
| --- | --- |
| Total Length (bp) | 5,033,995 |
| No. of contigs | 69 |
| GC Content (%) | 55.9% |
| N50 | 219,705 |
| Gap Ratio (%) | 0.0% |
| No. of CDSs | 4,621 |
| No. of rRNA | 33 |
| No. of tRNA | 110 |
| No. of CRISPRs | 0 |
| Coding Ratio (%) | 86.8% |

**ST 5:** Summary of short-chain C4-AHL produced by AG129 via HPLC/MS, C4-AHL in nM concentration.

| **RT** |  | **AG129 (nM)** | **AG129*easI* (nM)** | **Medium (nM)** |
| --- | --- | --- | --- | --- |
| 7,024 | C4 | 1128,68733 | 4,159444 | 28,5869276 |
| 12,674 | C6 | 6,02050566 | 0 | 0 |
| 16,461 | OHC10 | 2,29775053 | 2,24440973 | 3,07115685 |
| 19,667 | OHC12 | 4,35922997 | 4,86031679 | 5,55370866 |

**ST 6:** Concise summary of the proteins that showed differential expression in the secretome analysis, focusing on those with a log2 fold change greater than one.

| **AG129 upregulated while 129easI down regulated** | | |
| --- | --- | --- |
| **Protein ID** | **JGI/IMG** | **log2 fold change** |
| Ga0485092_69_112059_114599 | type VI secretion system secreted protein VgrG | -7,28 |
| Ga0485092_69_127124_128113 | hypothetical protein | -5.94 |
| Ga0485092_07_163328_168631 | hypothetical protein | -5.33 |
| Ga0485092_69_105519_109889 | RHS repeat-associated protein | -5.07 |
| Ga0485092_69_128707_129189 | type VI secretion system secreted protein Hcp | -3.89 |
| Ga0485092_08_119648_120166 | Cu-Zn family superoxide dismutase | -3.24 |
| Ga0485092_57_76365_77624 | hypothetical protein | -3.21 |
| Ga0485092_11_143986_147993 | filamentous hemagglutinin | -3.15 |
| Ga0485092_57_82876_84006 | hypothetical protein | -3.03 |
| Ga0485092_69_140769_141263 | hypothetical protein | -2.86 |
| Ga0485092_43_3_3479 | adhesin HecA-like repeat protein | -2.64 |
| Ga0485092_16_25564_26061 | large subunit ribosomal protein L10 | -2.22 |
| **Ga0485092_39_1104_5966** | **filamentous hemagglutinin** | **-2.16** |
| **Ga0485092_40_3_3512** | **adhesin HecA-like repeat protein** | **-1.98** |
| Ga0485092_07_78307_79605 | enolase | -1.86 |
| Ga0485092_21_1212_2048 | pimeloyl-ACP methyl ester carboxylesterase | -1.81 |
| Ga0485092_03_256749_257024 | ABC-type uncharacterized transport system substrate-binding protein | -1.75 |
| Ga0485092_05_149019_149288 | hypothetical protein | -1.72 |
| Ga0485092_16_28804_29772 | elongation factor Tu | -1.67 |
| Ga0485092_28_299976_301142 | succinyl-CoA synthetase beta subunit | -1.58 |
| Ga0485092_67_41804_42421 | hyperosmotically inducible protein | -1.52 |
| Ga0485092_01_138643_139755 | outer membrane pore protein C | -1.49 |
| Ga0485092_05_206305_206679 | translation elongation factor TU | -1.45 |
| Ga0485092_25_33964_34710 | outer membrane protein | -1.4 |
| Ga0485092_01_240522_241805 | long-chain fatty acid transport protein | -1,32 |
| Ga0485092_09_50740_51078 | cell shape-determining protein MreC | -1,25 |
| Ga0485092_01_247630_248976 | hypothetical protein | -1,13 |
| Ga0485092_08_19223_19567 | osmotically inducible lipoprotein OsmE | -1,07 |
| Ga0485092_01_262219_262401 | hypothetical protein | -1,04 |
| Ga0485092_01_30251_31303 | class I fructose-bisphosphate aldolase | -1,04 |
| Ga0485092_01_110738_111127 | lipoprotein Spr | -1 |
| Ga0485092_28_283215_283967 | 2,3-bisphosphoglycerate-dependent phosphoglycerate mutase | -0,974 |
| **129easI upregulated while AG129 down regulated** | | |
| Ga0485092_14_41426_44242 | isoleucyl-tRNA synthetase | 5.71 |
| Ga0485092_16_3839_4111 | DNA-binding protein HU-alpha | 3.84 |
| Ga0485092_15_60288_60683 | small subunit ribosomal protein S6 | 3.76 |
| Ga0485092_26_73115_73435 | type 1 fimbria pilin | 3.54 |
| Ga0485092_23_55833_58082 | iron complex outermembrane receptor protein | 3.4 |
| Ga0485092_16_25132_25497 | large subunit ribosomal protein L7/L12 | 3.14 |
| Ga0485092_05_200211_201029 | FKBP-type peptidyl-prolyl cis-trans isomerase FkpA | 2.69 |
| Ga0485092_08_125501_125968 | outer membrane lipoprotein SlyB | 2.41 |
| Ga0485092_06_43520_43729 | CspA family cold shock protein | 2.3 |
| Ga0485092_09_58822_59079 | phosphocarrier protein HPr | 2.26 |
| Ga0485092_03_153120_154418 | trigger factor | 2.03 |
| Ga0485092_26_49717_50133 | flagellar basal-body rod protein FlgB | 2.01 |
| Ga0485092_08_55852_56673 | iron complex transport system substrate-binding protein | 1.75 |
| Ga0485092_18_10241_10594 | large subunit ribosomal protein L18 | 1.74 |
| Ga0485092_15_15536_15829 | chaperonin GroES | 1.73 |
| Ga0485092_14_34353_36266 | molecular chaperone DnaK | 1.71 |
| Ga0485092_26_45740_46522 | flagellar basal-body rod protein FlgG | 1.69 |
| Ga0485092_23_78031_78882 | elongation factor Ts | 1.68 |
| Ga0485092_26_49309_49713 | flagellar basal-body rod protein FlgC | 1.67 |
| Ga0485092_08_97463_98299 | polar amino acid transport system substrate-binding protein | 1.56 |
| Ga0485092_08_15779_16273 | Spy/CpxP family protein refolding chaperone | 1.54 |
| Ga0485092_04_8383_9006 | thiol:disulfide interchange protein DsbA | 1.5 |
| Ga0485092_57_51334_52443 | branched-chain amino acid transport system substrate-binding protein | 1.5 |
| Ga0485092_23_87527_88021 | outer membrane protein | 1.43 |
| Ga0485092_09_60892_61401 | PTS system glucose-specific IIA component | 1.41 |
| Ga0485092_69_47357_48988 | oligopeptide transport system substrate-binding protein | 1.41 |
| Ga0485092_41_22382_23491 | outer membrane pore protein C | 1.4 |
| Ga0485092_01_259962_260135 | hypothetical protein | 1.29 |
| Ga0485092_42_25656_26117 | acetyl-CoA carboxylase biotin carboxyl carrier protein | 1.25 |
| Ga0485092_01_263622_264377 | hypothetical protein | 1.24 |
| Ga0485092_26_48587_49297 | flagellar basal-body rod modification protein FlgD | 1.24 |
| Ga0485092_03_186769_187029 | large subunit ribosomal protein L31 | 1.21 |
| Ga0485092_41_334670_335731 | YVTN family beta-propeller protein | 1.15 |
| Ga0485092_01_212319_213101 | histidine transport system substrate-binding protein | 1.14 |
| Ga0485092_42_132710_133255 | osmotically inducible protein OsmY | 1.14 |
| Ga0485092_26_46534_47289 | flagellar basal-body rod protein FlgF | 1.07 |
| Ga0485092_28_61413_62192 | uncharacterized SAM-binding protein YcdF (DUF218 family) | 3,32 |
| Ga0485092_03_247859_248380 | minor fimbrial subunit | 2,7 |
| Ga0485092_08_172400_173344 | hypothetical protein | 1,04 |
| Ga0485092_26_40237_41190 | flagellar hook-associated protein 3 FlgL | 1 |
| Ga0485092_02_112435_113667 | flagellar hook-length control protein FliK | 0,918 |
